# Mesenchymal to epithelial transition driven by canine distemper virus infection of canine histiocytic sarcoma cells contributes to a reduced cell motility in vitro

**DOI:** 10.1111/jcmm.15585

**Published:** 2020-07-06

**Authors:** Federico Armando, Matteo Gambini, Attilio Corradi, Kathrin Becker, Katarzyna Marek, Vanessa Maria Pfankuche, Ahmed Elmonastir Mergani, Graham Brogden, Nicole de Buhr, Maren von Köckritz‐Blickwede, Hassan Y. Naim, Wolfgang Baumgärtner, Christina Puff

**Affiliations:** ^1^ Department of Pathology University of Veterinary Medicine Hannover Hannover Germany; ^2^ Pathology Unit Department of Veterinary Medicine University of Parma Parma Italy; ^3^ Dipartimento di Medicina Veterinaria (DIMEVET) Universitá degli Studi di Milano Lodi Italy; ^4^ Department of Physiological Chemistry University of Veterinary Medicine Hannover Hannover Germany; ^5^ TWINCORE Centre for Experimental and Clinical Infection Research Hannover Hannover Germany Germany; ^6^ Research Center for Emerging Infections and Zoonoses (RIZ) University of Veterinary Medicine Hannover Hannover Germany

**Keywords:** 3D modelling, canine distemper virus, canine histiocytic sarcoma, DH82, mesenchymal to epithelial transition, viral oncolysis

## Abstract

Sarcomas especially of histiocytic origin often possess a poor prognosis and response to conventional therapies. Interestingly, tumours undergoing mesenchymal to epithelial transition (MET) are often associated with a favourable clinical outcome. This process is characterized by an increased expression of epithelial markers leading to a decreased invasion and metastatic rate. Based on the failure of conventional therapies, viral oncolysis might represent a promising alternative with canine distemper virus (CDV) as a possible candidate. This study hypothesizes that a CDV infection of canine histiocytic sarcoma cells (DH82 cells) triggers the MET process leading to a decreased cellular motility. Immunofluorescence and immunoblotting were used to investigate the expression of epithelial and mesenchymal markers followed by scratch assay and an invasion assay as functional confirmation. Furthermore, microarray data were analysed for genes associated with the MET process, invasion and angiogenesis. CDV‐infected cells exhibited an increased expression of epithelial markers such as E‐cadherin and cytokeratin 8 compared to controls, indicating a MET process. This was accompanied by a reduced cell motility and invasiveness. Summarized, these results suggest that CDV infection of DH82 cells triggers the MET process by an increased expression of epithelial markers resulting in a decreased cell motility in vitro.

## 1 INTRODUCTION

Malignant neoplasms represent nowadays one of the most common causes of death in humans and companion animals due to their often rapid and lethal progression.^1^, ^2^ Histiocytic sarcomas (HS) are malignant tumours that may occur in a systemic or localized form in both humans and dogs, with a comparable poor prognosis.^3^, ^4^, ^5^, ^6^ Therefore, dogs may represent an interesting translational model for this neoplastic disease due to the higher prevalence of HS in the canine species than in humans.^3^ Patients with HS often have short survival times due to the high metastatic rate of this neoplasm which is furthermore characterized by a limited and ineffective response to conventional therapies including surgery, chemo‐ and radiotherapy.^4^, ^5^ Therefore, novel and more effective approaches against this neoplasia are highly demanded. Since the beginning of the twentieth century the idea to use an oncolytic virus against neoplastic cells took place^6^ considered the ability of several viruses to preferentially infect and destroy cancer cells with direct and indirect mechanisms.^7^, ^8^ In human medicine, several oncolytic viruses (OVs) are currently used in clinical trials, including adenovirus, herpes simplex virus, vaccinia virus, reovirus, Seneca Valley virus and measles virus (MV).^9^ The latter is a morbillivirus that belongs to the *Paramyxoviridae* family.^9^ Another morbillivirus, closely related to MV, is canine distemper virus (CDV), which shares many common features with the first, including the ability to infect and induce apoptosis in lymphoid cells.^10^, ^11^ Therefore, CDV represents a promising candidate for future applications as an oncolytic virus for canine hematopoietic tumours.

CDV demonstrated the ability to persistently infect canine histiocytic sarcoma cells (DH82 cells), influencing the expression of reversion‐inducing cysteine‐rich protein with Kazal motifs (RECK), matrix metalloproteinases (MMP) −2 and −9 and tissue inhibitors of matrix metalloproteinases (TIMP) −1 and −2,^12^ altering cortactin distribution within the cytoskeleton,^13^ and reducing the expression of genes known to interfere with angiogenesis.^14^ Taken together, all these findings provide a robust basis to confirm CDV as a promising oncolytic virus for HS in dogs and use it as a model for the corresponding human disease.

During the last decade, the knowledge about factors influencing the biological behaviour of malignant neoplasms constantly increased. Specifically, the transition of cells from an epithelial to a mesenchymal state (EMT process) has been extensively studied and validated as one of the major features correlated to invasiveness and metastatic rate of carcinomas.^15^, ^16^ In contrast, the reverse transition known as mesenchymal to epithelial transition (MET process) came into the research focus only recently.^17^ The latter process is characterized by the expression of markers typical of epithelial cells in sarcomas, which is often linked with a favourable clinical outcome and a better prognosis.^17^ For example, in human synovial sarcoma, the epithelial cell markers E‐cadherin and β‐catenin are considered as potential positive prognostic factors.^18^ Additionally, longer survival time has been associated with E‐cadherin expression both at protein and mRNA level in a subset of human leiomyosarcomas.^19^ E‐cadherin has also been implicated as a tumour suppressor due to its protective role against epithelial to mesenchymal transition (EMT) at the primary site in carcinomas.^20^ The MET process in sarcomas is characterized by an increased expression of classical epithelial markers, whereas the classical mesenchymal markers still predominate in the tumour cells therefore determining the so‐called ‘metastable phenotype’.^17^, ^20^, ^21^ Typical epithelial‐like markers include proteins such as cytokeratin, CD44, CD34, β‐catenin and E‐cadherin.^17^ N‐cadherin, vimentin, desmin and alpha‐smooth muscle actin (α‐SMA) are considered among the typical mesenchymal markers.^17^

The hypothesis underlying the aim of this study is that a persistent infection of histiocytic sarcoma cells (DH82 cells) with CDV, strain Onderstepoort (CDV‐Ond), triggers the MET process by increasing the expression of epithelial markers, resulting in a less invasive phenotype with decreased motility of the neoplastic cells.^13^

## 2 MATERIALS AND METHODS

### 2.1 Cell culture

Non‐infected DH82 cells, a permanent canine histiocytic sarcoma cell line, were obtained from the European Collection of Authenticated Cell Cultures (ECACC No. 94062922). Persistently CDV (strain Onderstepoort)‐infected DH82 cells (DH82Ond pi) were produced as previously described.^12^ Cells were cultured in minimal essential medium (MEM) with Earle's salts (PAA, Cölbe, Germany) supplemented with 10% foetal calf serum (PAA), 1% penicillin/streptomycin (PAA) and 1% non‐essential amino acids (Sigma‐Aldrich, Taufkirchen, Germany). Culture flasks were kept at standard conditions (37°C with 5% CO_2_ in a water saturated atmosphere).

### 2.2 Morphological analysis using phase contrast microscopy

The morphology of non‐infected DH82 and DH82Ond pi cells was analysed using a phase contrast microscope (Olympus IX‐70, Olympus Optical Co. GmbH) equipped with an Olympus DP72 camera and Olympus cell sense standard software version 2.3. Cells were observed at 6 hours, 12 hours, 24 hours, 36 hours, 48 hours, 60 hours, 3 days, 4 days, 5 days, 6 days and 7 days after seeding. Afterwards, cells were counted according to their morphology and grouped in 4 different categories: round‐shaped, triangle‐shaped, cigar‐shaped and slender (supplementary material and Figure S1).

### 2.3 Cumulative population doubling assay

The population doubling assay was performed as previously described^13^ by evaluating non‐infected DH82 cells and DH82Ond pi cells over 14 weeks. Briefly, cells were seeded into 75‐cm^2^ flasks (Nunc GmbH & Co. KG, Thermo Scientific, Langenselbold, Germany) and counted at every weekly passage over 14 weeks. Population doubling was determined using the following population doubling (PD) formula: PD = log10 (cells harvested—initial cell number)/log2.^22^ Then, the cumulative population doubling was determined by adding the PD of every weekly passage to the previous one.

Statistical analysis as well as graphical visualization was performed using GraphPad Prism version 8.0.1 for Windows (GraphPad Software, La Jolla California USA, www.graphpad.com). The values were analysed with non‐parametric Wilcoxon–Mann–Whitney two‐sample test, setting the significance level at *P* ≤ .05.

### 2.4 Microarray data analysis using a manually generated list of gene symbols related to MET and invasiveness

Data of a previously published microarray dataset of non‐infected DH82 and DH82Ond pi cells (ArrayExpress; http://www.ebi.ac.uk/arrayexpress; accession number E‐MTAB‐3942)^13^, ^14^, ^23^ were used to evaluate the potential influence of a persistent CDV infection on genes associated with EMT/MET and cellular motility. In a hypothesis‐driven approach, the present study focused on a manually generated list of selected genes associated with EMT/MET, invasion and angiogenesis. Selected gene symbols were based on previously published lists,^13^, ^14^ which were further modified and extended (Table S1). According to the biological function of the corresponding protein(s), each selected gene symbol was assigned to ‘EMT/MET’ and/or ‘invasion and angiogenesis’ functional group. Genes were considered as differentially expressed between non‐infected DH82 and DH82Ond pi cells combining a fold change (FC) filter (FC ≥ 1.5 or ≤−1.5)^24^ with a statistical significance filter (Mann–Whitney *U* test; *P* ≤ .05).

### 2.5 Immunofluorescence

An initial qualitative immunohistochemical screening of the expression of E‐cadherin, β‐catenin, cytokeratin 8 (CK8), vimentin and N‐cadherin was performed on formalin‐fixed paraffin‐embedded (FFPE) cell pellets of both non‐infected and persistently CDV‐infected DH82 cells (Supplementary material and Table S2).

Non‐infected DH82 and DH82Ond pi cells were seeded at a density of 0.03 * 10^6^ cells/0.33 cm^2^ into 96 Microwell Nunc plates (Nunc GmbH & Co. KG, Thermo Scientific). All the immunostainings were performed in triplicates with negative controls in duplicates. 3 days after seeding, cells were fixed with 4% paraformaldehyde and immunofluorescence was performed according to a 2 days protocol with minor variations.^25^ To verify the persistent CDV infection state of DH82Ond pi cells, an immunolabelling with an anti‐CDV nucleoprotein (CDV‐NP) antibody (clone D110; kindly provided by Prof. Dr A. Zurbriggen, University of Bern, Switzerland) was performed as previously described.^13^ Furthermore, cells were immunolabelled for E‐cadherin, β‐catenin and cytokeratin 8 as epithelial markers, and for vimentin and N‐cadherin as mesenchymal markers. All details regarding the aforementioned antibodies are listed in Table 1. For negative controls, the first antibody was replaced with rabbit serum, Balb/c ascitic fluid or goat serum, respectively, at corresponding protein concentrations. For all the aforementioned markers, the percentage of immunopositive cells was determined for each group (non‐infected DH82 cells and DH82Ond pi cells) by counting 5 evenly distributed fields per well, taking pictures at a 400× magnification using a fluorescence microscope (Olympus IX‐70, Olympus Optical Co. GmbH) equipped with an Olympus DP72 camera and Olympus cell sense standard software version 2.3. The analysed pictures were taken from areas of different confluence (low, medium, high) for each marker and for both persistently CDV‐infected and non‐infected DH82 cells. Besides the determination of the overall percentage of positive cells for each marker, the number of positive cells based on the cell shape (round, spindle or multinucleated giant cells) as well as the number of positive cells based on the intracellular localization (membranous, membranous to cytoplasmic, diffusely cytoplasmic, focally cytoplasmic) was additionally evaluated. Detailed descriptions of the cell shape and of the intracellular distribution pattern are available as supplementary material. Statistical analysis as well as graphical visualization was carried out using GraphPad Prism version 8.0.1 for Windows (GraphPad Software, La Jolla California USA, www.graphpad.com. The values were analysed with Student's *t* test, setting the significance level at *P* ≤ .05.

**Table 1:** Details of the antibodies and the lectin used for the immunostaining including primary antibody, host species, clonality, blocking serum, dilution of primary antibody and secondary antibody

Primary antibody	Host species, clonality	Serum blocking	Dilution	Secondary antibody (1:200)
Beta catenin (Sicgen)	Goat, polyclonal	Horse serum	1:100	DaG‐Cy3
Cytokeratin 8 (Invitrogen)	Rabbit, polyclonal	Goat serum	1:200	GaR‐Cy3/GaR‐AF 488^a^
E‐cadherin (BD transduction laboratory)	Mouse, monoclonal clone 36/E‐Cadherin (RUO)	Goat serum	1:200	GaM‐Cy2/GaM AF488^b^
N‐cadherin (Proteintech)	Rabbit, polyclonal	Goat serum	1:100	GaR‐Cy3
Vimentin (Dako)	Mouse, monoclonal clone V9	Goat serum	1:100	GaM‐Cy2/GaM AF488^b^
CDV‐NP (University of Bern)	Mouse, monoclonal clone D110	Goat serum	1:100	GaM‐Cy2
WGA – AF633 conjugated (Invitrogen, California, USA)	None	n/a	1:20	n/a

Abbreviations: CDV‐NP, canine distemper virus nucleoprotein; DaG‐Cy3, donkey anti goat cyanine 3‐conjugated; GaM‐AF488, goat anti mouse Alexa Fluor 488‐conjugated; GaM‐Cy2, goat anti mouse cyanine 2‐conjugated; GaR‐AF488, goat anti rabbit Alexa Fluor 488‐conjugated, GaR‐Cy3, goat anti rabbit cyanine 3‐conjugated; n/a, non applied or non applicable, WGA – AF633, wheat germ agglutinin – Alexa Fluor 633 conjugated.

^a^ GaR‐Cy3 was used for single‐labelling and GaR‐Cy2 for double‐labelling stains.

^b^ GaM‐Cy2 was used for single‐labelling and GaM‐AF488 for double‐labelling stains.

### 2.6 Laser scanning confocal microscopy and 3D reconstruction

E‐cadherin, cytokeratin 8 and vimentin were further analysed with laser scanning confocal microscopy to allow a better characterization of the intracellular localization of these markers. Firstly, confocal microscopy was used to evaluate cells stained with single‐labelling immunofluorescence for E‐cadherin and cytokeratin 8. Then, to allow a better characterization of the spatial distribution of E‐cadherin, cytokeratin 8 and vimentin within the cell volume, double‐labelling immunofluorescence was performed combining each marker with wheat germ agglutinin (WGA), which was used to stain cell membrane and Golgi apparatus. Double staining immunolabellings with WGA were performed as previously described in^26^ with variations, according to the protocol available as supplementary material. Details about the antibodies are listed in Table 1. Cells were analysed using a Leica TCS SP5 AOBS confocal inverted‐base fluorescence microscope (Leica Microsystems, Bensheim, Germany) with a conventional galvanometer scanner of the Leica SP5 II tandem scanning system and the Leica Application Suite Advanced Fluorescent Lite 2.0.2 build 2038 (Leica, Biberach, Germany). The microscope was equipped with 3 lasers [405 DIODE (415/2471), ARGON (500/2571) and HeNe 633 (639/2715)], and with HCX PL APO ×40 0.75‐1.25 and HCX PL APO ×63 0.75‐1.25 oil immersion objectives used for the evaluation of the single and double‐labelling immunofluorescence, respectively. Settings for each marker and each stain (ie single‐ or double‐labelling) were adjusted using the corresponding appropriate controls. For the single‐labelling stains, images were analysed using Leica LAS 244 AF software (version 2.7.3). For the 3D reconstructions of the double‐labelling immunofluorescences, z‐stacks were collected and analysed with LAS X 3D version 3.1.0 software from Leica. The specific number of z‐stack frames (0.13 µm steps) ranged from 65 to 151 (the specific number of frames for each z‐stack is reported in the caption of the corresponding figure). For each z‐stack set, the background was set to black by standard software settings. Subsequently, top view and section view of the 3D reconstructions were created for each staining. Section view allowed to analyse protein localization within the cells.

### 2.7 Immunoblotting

Immunoblotting of non‐infected and persistently CDV‐infected DH82 cells was comparatively carried out in three independent samples for each cell type, as formerly described.^27^ Following cell lysis, the correct amount of each sample required for the analysis was calculated based on the protein concentration as determined applying the Bradford method. Immunoblotting was performed using as primary antibodies polyclonal anti‐β‐catenin (1:500, Sicgen) and anti‐CK8 (1:2000, Invitrogen), and monoclonal anti‐β‐actin (1:200, Santa Cruz, Dallas, USA) and anti‐E‐cadherin (1:200, BD transduction laboratory) antibodies. To ensure that the observed differences actually reflected differences in the protein expression, β‐actin was set as a house‐keeping protein to allow the investigation of the relative expression of β‐catenin, E‐cadherin and CK8 following densitometric analysis and using β‐actin for normalization. Therefore, the differential expression of the β‐actin gene and protein between non‐infected and persistently CDV‐infected DH82 cells was evaluated with microarray data and densitometry of Western blot bands, respectively. Specifically, for the microarray data analysis a combination of a fold change (FC) filter (FC ≥ 1.5 or ≤−1.5) with a statistical significance filter (Mann–Whitney *U* test; *P* ≤ .05) was applied. Then, for each cell lysate the protein amount of β‐catenin, E‐cadherin and CK8 was quantified densitometrically and obtained results were shown as a ratio with the corresponding amount of β‐actin. Statistical analysis was carried out using GraphPad Prism version 8.0.1 for Windows (GraphPad Software, La Jolla California USA, www.graphpad.com). The overall values of β‐actin as well as the values of the ratios of β‐catenin, E‐cadherin and CK8 to β‐actin were analysed with unpaired *t* test, setting the significance level at *P* ≤ .05. Further information is available as supplementary material.

### 2.8 Scratch assay and invasion assay

For the scratch assay, non‐infected DH82 and DH82Ond pi cells were seeded in 3 wells each at a density of 0.3 * 10^6^ cells/1.9 cm^2^ into 24 well/plates (Nunc GmbH & Co. KG, Thermo Scientific) with 1 mL of minimal essential medium (MEM) with Earle's salts (PAA, Cölbe, Germany) supplemented with 10% foetal calf serum (PAA), 1% penicillin/streptomycin (PAA) and 1% non‐essential amino acids (Sigma‐Aldrich, Taufkirchen, Germany). Three days after seeding and cultivation under standard conditions (37°C with 5% CO_2_ in a water saturated atmosphere) cells reached 99% of confluence. The monolayer of cells was then scratched in a straight line with a p1000 pipette tip. Medium and cellular debris were then slowly removed, and fresh culture medium was gently added. Pictures were taken at the same position for every time point using a phase contrast microscope (OlympusIX‐70, Olympus Optical Co. GmbH) equipped with an Olympus DP72 camera and Olympus cell sense standard software version. The aforementioned software was used to define and calculate the cell‐free area of the scratch. Following the scratch (time point 0), the measurement of the wound area was performed after 6 and 24 hours. The percentage of scratch closure was calculated according to the following formula: (Area T_0_ – Area T_x_)/Area T_0_.

For the invasion assay, non‐infected DH82 and DH82Ond pi cells were seeded in 6 wells each at a density of 0.04 * 10^6^ cells/0.33 cm^2^ into 96 well/plates (Nunc GmbH & Co. KG, Thermo Scientific) with 200 µL of MEM with Earle's salts (PAA, Cölbe, Germany) supplemented with 10% foetal calf serum (PAA), 1% penicillin/streptomycin (PAA) and 1% non‐essential amino acids (Sigma‐Aldrich). After seeding, cells were incubated under standard conditions (37°C with 5% CO_2_ in a water saturated atmosphere). When the cells reached the desired confluence (80%‐100%) 3 days after seeding, the cell monolayer was scratched creating a linear wound with a p100 pipette tip. Medium and cellular debris were gently removed and replaced by Matrigel matrix (Corning, New York, USA) diluted at a concentration of 3 mg/ml in culture medium. After gelification of the Matrigel matrix following incubation at 37°C, 5% CO_2_ for 2 hours, the Matrigel layer was covered with 50 µL of MEM with Earle's salts (PAA, Cölbe, Germany) supplemented with 1% penicillin/streptomycin (PAA), and 1% non‐essential amino acids (Sigma‐Aldrich), and the plates were re‐incubated under standard conditions. Cell invasion through Matrigel was evaluated 6, 24 and 144 hours after gelification of the matrix. For each well, a picture was taken at the same position for every time point using a phase contrast microscope (Olympus IX‐70, Olympus Optical Co. GmbH) equipped with an Olympus DP72 camera and Olympus cell sense standard software version. Pictures were analysed with Fiji (ImageJ 1,52p)^28^ to define and calculate the cell‐free area of each picture. Specifically, after 8bit conversion and automatic adjustment of brightness/contrast of the picture followed by an automatic subtraction of background staining, the threshold was set to 0 and 194 and cell‐covered area was determined with the analyse particle function using an overlay mask. The percentage of cell‐free area was then calculated according to the following formula: 100 – cell‐covered area, with the latter corresponding to the percentage value automatically determined by Fiji (see also supplementary material). The percentage of variation of the cell‐free area was then calculated according to the following formula: (|Area T0 – Area Tx|)/Area T0 * 100, with Tx alternatively referring to the 6 hours or the 24 hours time point.

For both the scratch and the invasion assay, statistical analysis as well as graphical visualization was carried out using GraphPad Prism version 8.0.1 for Windows (GraphPad Software, La Jolla California USA, www.graphpad.com). The data obtained by the scratch assay and the invasion assay were compared between persistently infected and non‐infected cells using a two‐way ANOVA, setting the significance level at *P* ≤ .05.

## 3 RESULTS

### 3.1 Persistent CDV‐Ond infection of DH82 cells leads to morphological changes while growth features remain unaltered

The infection status of DH82Ond pi cells was assessed via immunofluorescence staining for CDV‐NP. While immunoreactivity for CDV‐NP of DH82Ond pi cells showed an average of 97.5% infected cells (median: 97.5%; range: 96%‐99%), all non‐infected DH82 cells were negative (Figure S2). The cumulative population doubling assay did not show any difference (*P* = .6347) between non‐infected and persistently CDV‐Ond infected DH82 cells (Figure S3). Interestingly, both non‐infected and persistently CDV‐infected DH82 cells displayed morphological changes dependent of the time point post‐seeding. DH82Ond pi cells exhibited an increased percentage of round cells starting at 3 days post‐seeding accompanied by a decrease of the others 3 morphological phenotypes (triangle, cigar and slender) as shown in Figure S4. From 5 to 7 days post‐seeding, round cells predominated among DH82Ond pi cells. Non‐infected DH82 cells showed a highly pleomorphic phenotype during the first 2 days post‐seeding with an increased presence of the triangle and cigar‐shaped phenotypes. From days 5 to 7 post‐seeding, there was a mild increase in the percentage of round cells but with a constant presence of the 3 other morphological phenotypes, resulting in a moderately to highly pleomorphic appearance of non‐infected cultures at all time points investigated (Figure S5). Summarized, these results highlighted that a persistent CDV infection of DH82 cells did not alter cell growth features but led to morphological changes. Assuming that morphological alterations of the cell shape are a feature of MET associated with changes in epithelial and mesenchymal markers; further investigations were performed to verify the occurrence of this phenomenon in non‐infected and persistently CDV‐Ond infected DH82 cells.

### 3.2 DH82Ond pi cells display an increased expression of epithelial markers on a protein level

**Figure 1:**
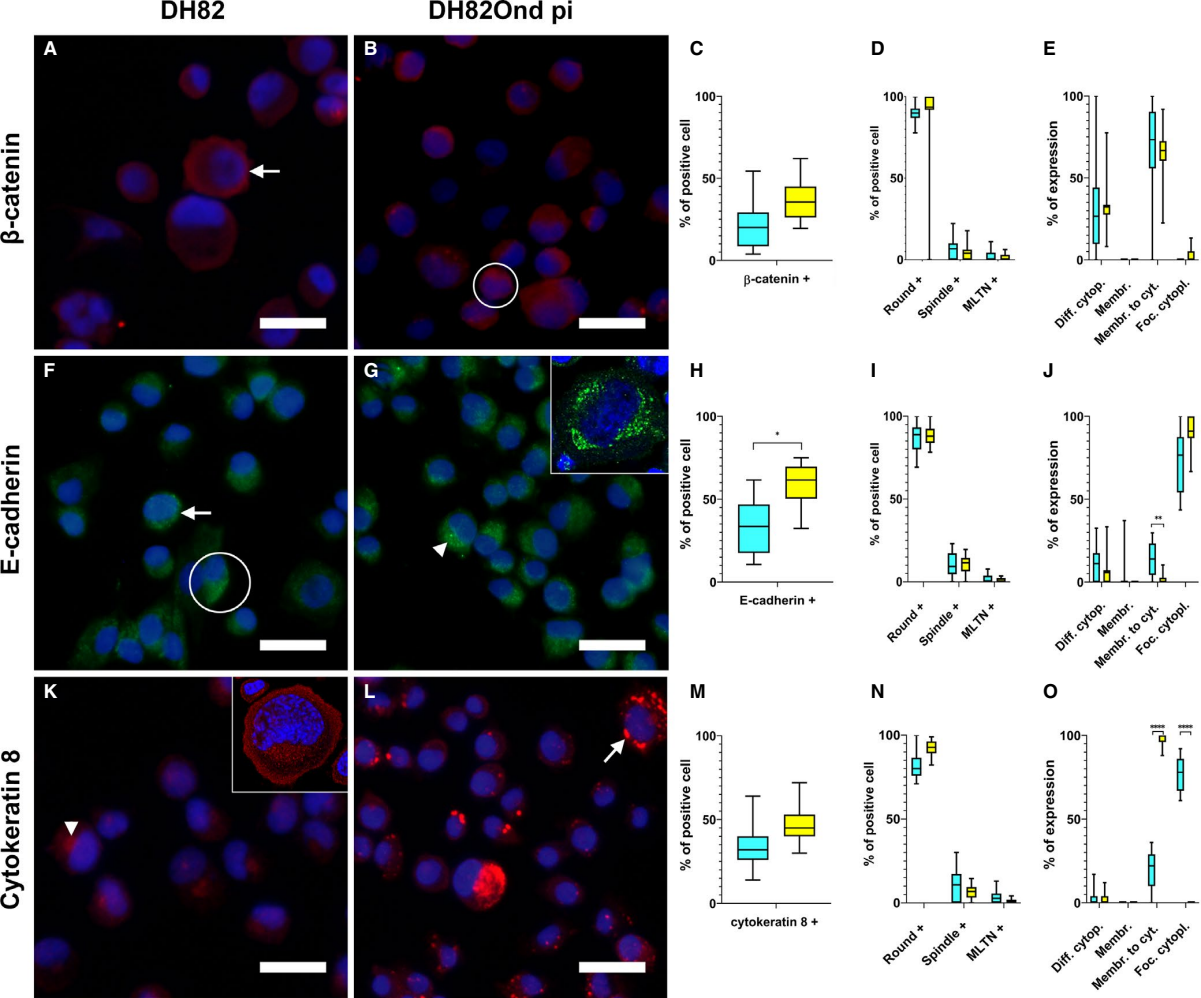
Immunofluorescence for epithelial markers in non‐infected (A, F, K) and persistently CDV‐infected DH82 cells (B, G, L). Nuclei were labelled with bisbenzimide (blue). Graphs show immunofluorescence results for epithelial markers analysed in non‐infected and persistently CDV‐infected DH82 cells according to overall percentage of positive cells for each marker (C, H, M), cellular morphology (D, I, N) and intracellular localization (E, J, O). Staining for β‐catenin (Cy3, red) revealed that both non‐infected (A) and persistently CDV‐infected DH82 cells (B) expressed this protein, with an intracellular distribution pattern mainly ranging from ‘membranous to cytoplasmic’ (A, arrow) to diffusely cytoplasmic (B, encircled). β‐catenin expression was increased in DH82Ond pi compared to non‐infected controls, but did not reach statistical significance (C). No differences in β‐catenin expression were detected neither according to cell morphology (D) nor in intracellular distribution (E). Staining for E‐cadherin (Cy2, green) showed the expression of this protein in both non‐infected (F) and DH82Ond pi cells (G), with an intracellular distribution pattern ranging from ‘membranous to cytoplasmic’ (A, arrow) to diffuse (A, encircled) and focal cytoplasmic (B, arrowhead), as confirmed by laser scanning confocal microscopy (G, insert). A significantly higher number of DH82Ond pi cells expressed E‐cadherin compared to non‐infected controls (H). The expression of E‐cadherin did not differ between non‐infected and infected cells based on different morphologies (I). Non‐infected DH82 cells showed a significantly higher ‘membranous to cytoplasmic’ expression of E‐cadherin compared to DH82Ond pi cells (J). Staining for cytokeratin 8 (Cy3, red) demonstrated that both non‐infected (K) and persistently CDV‐infected DH82 cells (L) expressed this protein, with an intracellular distribution pattern mainly ranging from focal cytoplasmic (K, arrowhead) as confirmed by laser scanning confocal microscopy (K, insert), to ‘membranous to cytoplasmic’ (L, arrow). DH82Ond pi cells displayed an increased expression of cytokeratin 8, despite not reaching statistical significance (M). The expression of cytokeratin 8 displayed no significant differences between non‐infected and infected cells based on different morphologies (N). DH82Ond pi cells showed a significantly higher ‘membranous to cytoplasmic’ expression of cytokeratin 8 while non‐infected controls exhibited a significantly higher focal cytoplasmic expression (O). Bar = 20 µm, Box and whisker plots with median values, quartiles and maximum and minimum values. Significant differences (*P* ≤ .05, Student's *t* test) are labelled by asterisks. Graph legend: light blue = DH82 cells; yellow = DH82Ond pi cells; Diff. cytopl. = diffuse cytoplasmic; Membr. = membranous; Membr. to cyt. = membranous to cytoplasmic; Foc. cytopl. = focal cytoplasmic; MLTN+ = positive multinucleated tumour cells

Immunofluorescence of DH82Ond pi cells displayed an increased number of cells expressing β‐catenin (Figure 1A‐C; mean = 33%; median = 32%; range: 28%‐34%) compared to non‐infected controls (mean = 18%; median = 17%; range: 12%‐29%). However, this increase was not statistically significant (*P* = .0792). The expression of β‐catenin showed no significant differences between the different cell phenotypes and with respect to the localization within the cells (Figure 1D‐E).

**Figure 2:**
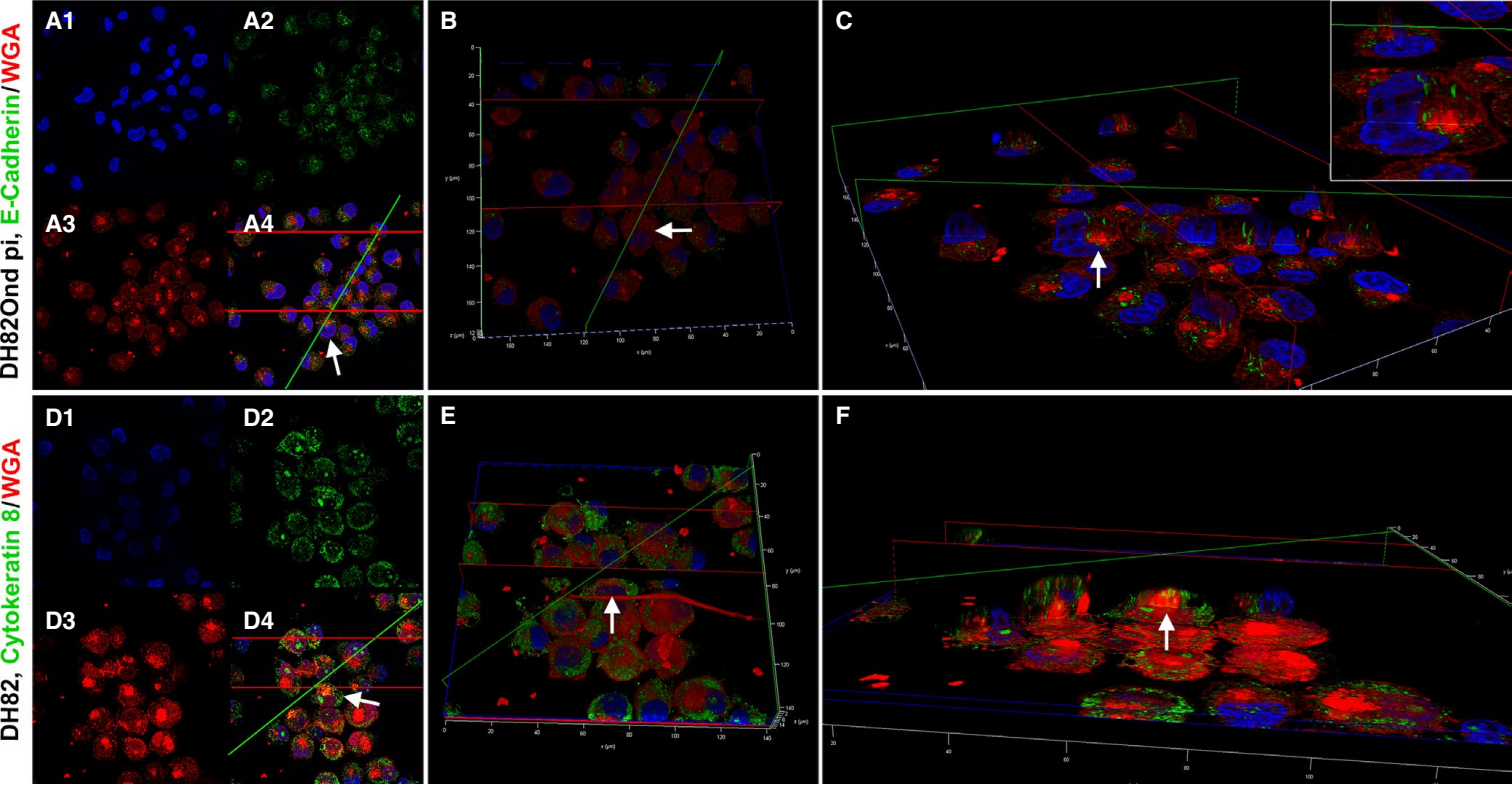
3D reconstructions from double‐labelling immunofluorescence of DH82Ond pi and non‐infected DH82 cells stained for E‐cadherin/WGA (A‐C) and cytokeratin 8/WGA (D‐F), respectively. A, 2D pictures of DH82Ond pi cells from z‐stacks obtained with laser scanning confocal microscopy. The single channel views are as follows: nuclei in blue, stained with bisbenzimide (A1); E‐cadherin in green, stained with Alexa Fluor 488 (A2); and WGA in red, conjugated with Alexa Fluor 633 (A3). In addition, the picture shows a merged 3 channels view (A4). B, Top view of the 3D reconstruction obtained from the z‐stack in (A), with the total cell volume represented by WGA in red. E‐cadherin was only occasionally expressed on the cell surface. C, Section view of the 3D reconstruction in (B), showing the model sectioned along the green and red planes to better display the E‐cadherin expression (green) within the cell volume (red). The protein was frequently localized in a focal area near the nucleus surrounding the Golgi apparatus (C, insert), mainly not extending to intermingle with the cell membrane. Each arrow represents the same cell from the z‐stack picture to the 3D section view. 3D reconstruction of DH82Ond pi double immunolabelling for E‐cadherin/ WGA was obtained by 135 z‐stack frames (0.13 µm steps). D, 2D pictures of non‐infected DH82 cells from z‐stacks obtained with laser scanning confocal microscopy. The single channel views are as follows: nuclei in blue, stained with bisbenzimide (D1), cytokeratin 8 in green, stained with Alexa Fluor 488 (D2); and WGA in red, conjugated with Alexa Fluor 633 (D3). In addition, the picture shows a merged 3 channels view (D4). E, Top view of the 3D reconstruction obtained from the z‐stack in (D), with the total cell volume represented by WGA in red. Cytokeratin 8 expression was also detected on the cell surface, supporting the evidence of a ‘membranous to cytoplasmic’ distribution pattern. F, Section view of the 3D reconstruction in (E), showing the model sectioned along the green and the red planes to better display cytokeratin 8 expression (green) within the cell volume (red). The protein mainly showed a focal cytoplasmic, variably sized immunopositive area near the nucleus, occasionally localizing above the Golgi apparatus and extending to intermingle with the cell membrane. Each arrow represents the same cells from the z‐stack picture to the 3D section view. 3D reconstruction of non‐infected DH82 double immunolabelling for cytokeratin 8/WGA was obtained by 154 z‐stack frames (0.13 µm steps)

Immunoreactivity for E‐cadherin (Figure 1F‐H) revealed a significantly (*P* = .0139) increased number of immunopositive DH82Ond pi cells (mean = 59%; median = 58%; range: 51%‐67%) compared to non‐infected controls (mean = 34%; median = 29%; range: 24%‐49%). When evaluated based on the different cell phenotypes, no statistical differences were detected between non‐infected and DH82Ond pi cells (Figure 1I). Non‐infected DH82 cells displayed a significantly (*P* = .0016) higher ‘membranous to cytoplasmic’ expression of E‐cadherin (Figure 1J). On the other hand, DH82Ond pi cells exhibited an increased focal cytoplasmic E‐cadherin expression compared to non‐infected controls, despite not reaching statistical significance (*P* = .0846). The unexpected E‐cadherin localization was firstly verified by laser scanning confocal microscopy of single‐labelling immunofluorescence stains (Figure 1G, insert). Furthermore, 3D reconstructions obtained from double‐labelling immunofluorescence combining E‐cadherin with WGA (Figure 2A‐C) confirmed that E‐cadherin within DH82Ond pi cells often localized in a cytoplasmic immunopositive focal area of variable size, which surrounded the Golgi apparatus without localizing within the latter. On the other hand, non‐infected controls showed also a membranous to cytoplasmic E‐cadherin expression pattern, confirming the results observed with single‐labelling immunofluorescence (Figure S6).

**Figure 3:**
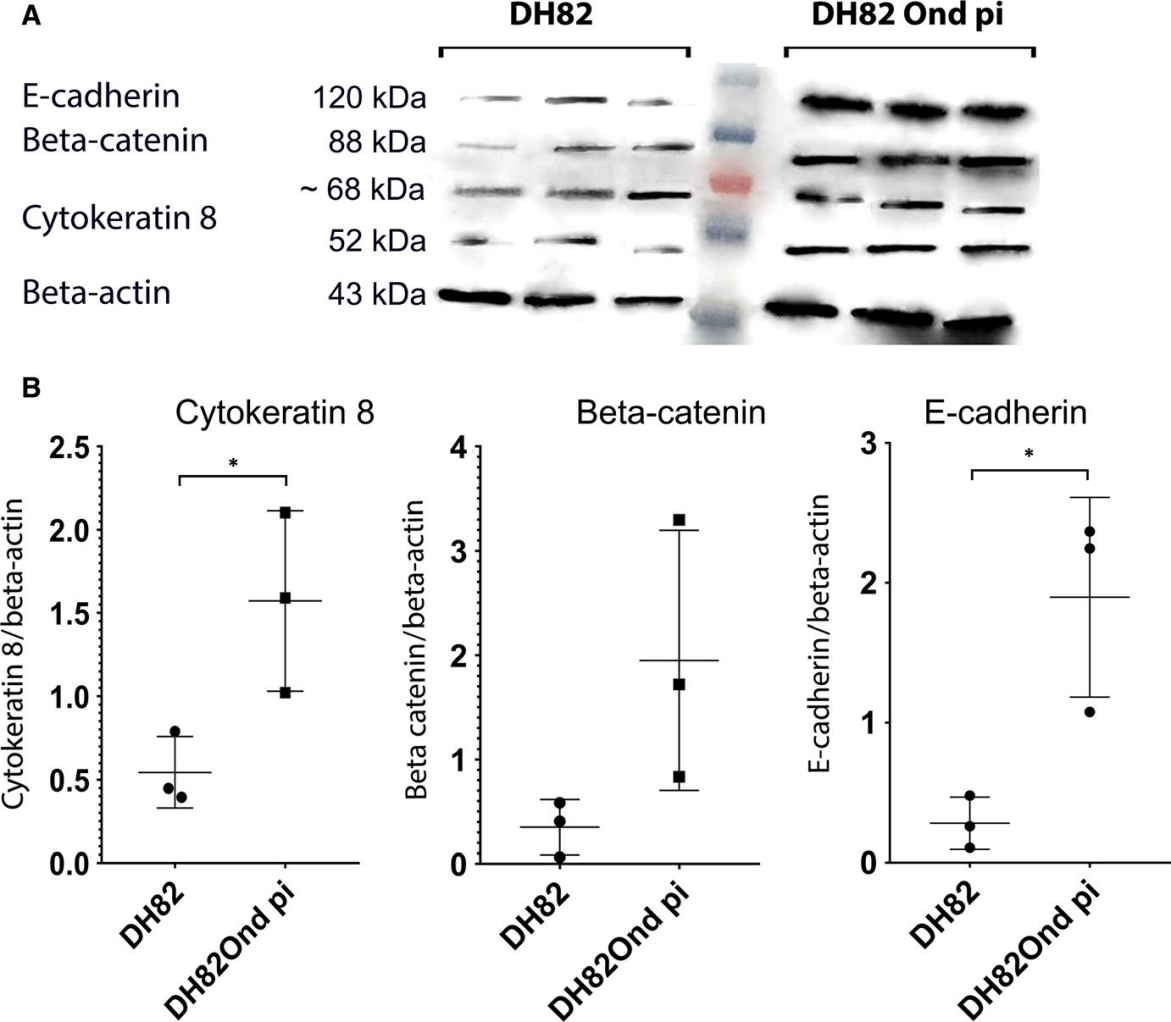
Immunoblotting with anti‐cytokeratin 8, anti‐β catenin, anti‐E‐cadherin and anti‐β‐actin antibodies revealed bands of 52, 88, 120 and 43 kD, respectively (A). Band sizes and intensities of the aforementioned markers were quantified densitometrically and compared as ratios to β‐actin, which was used as a house‐keeping protein considered that its expression displayed no differential expression between non‐infected and persistently CDV‐Ond infected DH82 cells at the gene level and with densitometric analyses. DH82Ond pi cells displayed a significantly higher amount of cytokeratin 8 and E‐cadherin compared to non‐infected controls, while β‐catenin failed to reach the level of significance. Both non‐infected and DH82Ond pi cells displayed bands of unspecific origin of ~68 kD (B). Dot plots show means and standard deviation. Statistically significant differences are labelled by asterisks (*P* ≤ .05)

Immunolabelling for cytokeratin 8 (Figure 1K‐M) lacked a significant difference (*P* = .0688) in the percentage of positive cells between DH82Ond pi (mean = 45%; median = 49%; range: 38%‐50%) and non‐infected controls (mean = 33%; median = 33%; range: 28%‐39%). Evaluation based on the different cell phenotypes revealed no statistically significant differences in the expression of cytokeratin 8 between DH82Ond pi and non‐infected controls (Figure 1N). However, DH82Ond pi cells displayed a significantly (*P* < .0001) higher ‘membranous to cytoplasmic’ expression of this protein compared to non‐infected controls (Figure 1O), while the diffuse cytoplasmic localization did not reach statistical significance (*P* = 0,8340). Interestingly, cytokeratin 8 displayed a focal cytoplasmic expression that was significantly (*P* < .0001) more often observed in non‐infected controls compared to persistently CDV‐infected DH82 cells (Figure 1O). Similarly to E‐cadherin, the focal cytoplasmic localization of this marker in non‐infected DH82 cells was initially confirmed by laser scanning confocal microscopy of single‐labelling immunofluorescence stains (Figure 1K, insert). Further analyses employing 3D reconstructions of double‐labelling immunofluorescence combining cytokeratin 8 and WGA (Figure 2D‐F) confirmed a focal cytokeratin 8 localization within non‐infected DH82 cells, revealing a variably sized and shaped immunopositive area located near the nucleus and the Golgi apparatus. Occasionally, also a membranous to cytoplasmic expression of the protein was detected in non‐infected controls. DH82Ond pi cells were analysed accordingly confirming a membranous to cytoplasmic expression of cytokeratin 8, which was frequently arranged in variably sized aggregates (Figure S6). Additional single‐labelling immunofluorescence pictures displaying the intracellular distribution of β‐catenin, E‐cadherin and cytokeratin 8 in non‐infected and persistently CDV‐infected DH82 cells at different confluences are available as Figure S7. In order to confirm the immunofluorescence results, an immunoblotting for all the investigated epithelial markers was performed (Figure 3A). Beta‐actin was used as a house‐keeping protein, lacking differential expression at both the gene (fold change: 1.06; p value < 0.001) and the protein level (*P* = .8446) between non‐infected and DH82Ond pi cells. DH82Ond pi cells contained a significantly higher amount of E‐cadherin (*P* = .0192) compared to non‐infected controls (Figure 3B). In addition, a similar higher amount of cytokeratin 8 expression was observed in DH82Ond pi cells compared to non‐infected controls (*P* = .0376). The β‐catenin expression was higher in DH82Ond pi compared to non‐infected DH82 cells (Figure 3B) although this increase did not reach statistical significance (*P* = .0956). Further investigations were performed to evaluate the expression of the typical mesenchymal markers (N‐cadherin, vimentin) in non‐infected and DH82Ond pi cells.

### 3.3 DH82Ond pi cells retain mesenchymal marker expression

**Figure 4:**
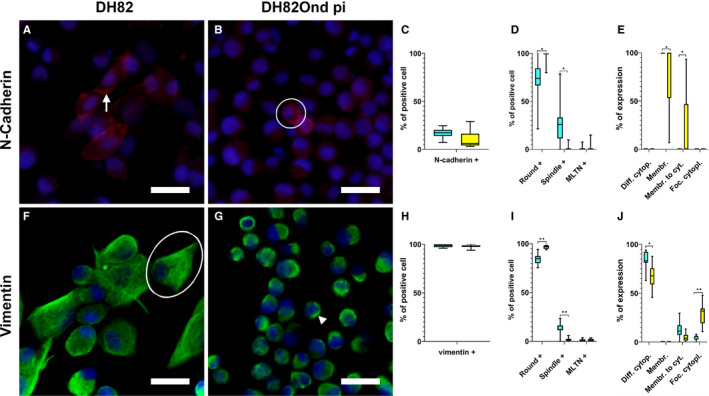
Immunofluorescence results for mesenchymal markers analysed in non‐infected (A, F) and persistently CDV‐infected DH82 cells (B, G) according to overall percentage of positive cells for each marker (C, H), cellular morphology (D, I) and intracellular localization (E, J). Nuclei were labelled with bisbenzimide (blue). Staining for N‐cadherin (Cy3, red) revealed that non‐infected (A) and persistently CDV‐infected (B) DH82 cells displayed an intracellular distribution pattern ranging from purely membranous (A, arrow) to ‘membranous to cytoplasmic’ (B, encircled). No significant differences in the number of cells expressing N‐cadherin were detected between non‐infected and persistently CDV‐infected DH82 cells (C). DH82Ond pi cells showed a significantly higher number of round cells expressing N‐cadherin while non‐infected controls displayed a significantly higher number of spindle‐shaped cells expressing this marker (D). Non‐infected DH82 cells exhibited a significantly higher expression of N‐cadherin in a membranous localization while persistently CDV‐infected DH82 cells showed a significantly increased ‘membranous to cytoplasmic’ expression of this marker (E). Both non‐infected (F) and DH82Ond pi cells (G) expressed vimentin (Cy2, green) with a diffuse cytoplasmic (F, encircled) to focal cytoplasmic (G, arrowhead) localization of this protein. Vimentin lacked a significant difference in the number of cells expressing the protein regardless of the infection state (H). The number of spindle‐shaped cells expressing vimentin was significantly higher in non‐infected DH82 cells while DH82Ond pi cells showed a significantly higher number of positive round‐shaped cells (I). On the other hand, DH82Ond pi cells exhibited a significantly increased focal cytoplasmic expression while non‐infected controls showed a significantly higher cytoplasmic expression of this protein (J). Bar = 20 µm, Box and whisker plots with median values, quartiles and maximum and minimum values. Significant differences (*P* ≤ .05, Student's *t* test) are labelled by asterisks. Graph legend: light blue = DH82 cells; yellow = DH82Ond pi cells; Diff. cytopl. = diffuse cytoplasmic; Membr. = membranous; Membr. to cyt. = membranous to cytoplasmic; Foc. cytopl. = focal cytoplasmic; MLTN+ = positive multinucleated tumour cells

Mesenchymal marker immunolabelling was analysed as shown in Figure 4.

The number of cells expressing N‐cadherin (Figure 4A‐C) did not differ significantly (*P* = .0975) between non‐infected (mean = 16%; median = 17%; range: 11%‐21%) and persistently CDV‐infected (mean = 8%; median = 7%; range: 5%‐13%) DH82 cells. Interestingly, non‐infected DH82 cells showed a significantly (*P* = .0185) higher number of spindle‐shaped cells expressing N‐cadherin (Figure 4D) compared to DH82Ond pi. On the other hand, in DH82Ond pi cells this protein was predominantly expressed in cells with a round morphology, which were significantly more abundant (*P* = .0156) compared to the same phenotype among non‐infected cells. Non‐infected cells demonstrated a significantly higher membranous N‐cadherin expression (Figure 4E) compared to DH82Ond pi cells (*P* = .0139). In contrast, persistently CDV‐infected DH82 cells exhibited a significantly higher ‘membranous to cytoplasmic’ expression of N‐cadherin compared to non‐infected controls (*P* = .0139).

**Figure 5:**
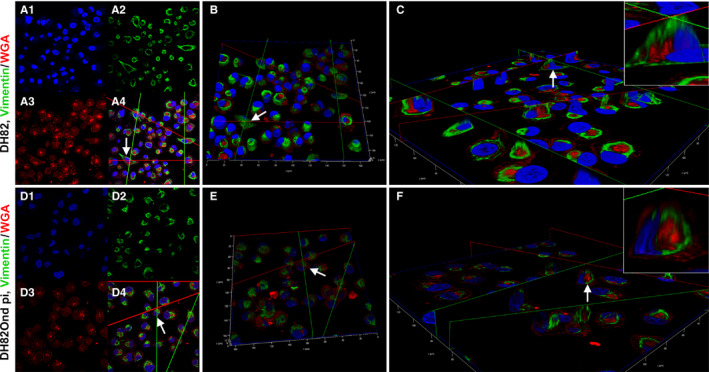
3D reconstructions from double‐labelling immunofluorescence of non‐infected DH82 (A‐C) and DH82Ond pi (D‐F) cells stained for vimentin and WGA. A, 2D pictures of non‐infected DH82Ond cells from z‐stacks obtained with laser scanning confocal microscopy. The single channel views are as follows: nuclei in blue, stained with bisbenzimide (A1); vimentin in green, stained with Alexa Fluor 488 (A2); and WGA in red, conjugated with Alexa Fluor 633 (A3). In addition, the picture shows a merged 3 channels view (A4). B, Top view of the 3D reconstruction obtained from the z‐stack in A, with the total cell volume represented by WGA in red. Especially within spindle‐shaped cells, vimentin was diffusely detected in the cytoplasm. C, Section view of the 3D reconstruction in (B), showing the model sectioned along the green and red planes to better display the vimentin expression (green) within the cell volume (red). Especially in the spindle‐shaped cells, the protein widely occupied the cytoplasm extending up to immediately below the cell membrane (C, insert). On the contrary, within the majority of round cells the vimentin signal was detected as a focal cytoplasmic expression. Each arrow represents the same cells from the z‐stack picture to the 3D section view. 3D reconstruction of non‐infected DH82 double immunolabelling for vimentin/WGA was obtained by 80 z‐stack frames (0.13 µm steps). D, 2D pictures of DH82Ond pi cells from z‐stacks obtained with laser scanning confocal microscopy. The single channel views are as follows: nuclei in blue, stained with bisbenzimide (D1); vimentin in green, stained with Alexa Fluor 488 (D2); and WGA in red, conjugated with Alexa Fluor 633 (D3). In addition, the picture shows a merged 3 channels view (D4). E, Top view of the 3D reconstruction obtained from the z‐stack in (D), with the total cell volume represented by WGA in red. Vimentin was expressed as a focal cytoplasmic staining (green), without extending to the cell borders. F, Section view of the 3D reconstruction in (E), showing the model sectioned along the green and red planes to better display the vimentin expression (green) within the cell volume (red). The cut section confirmed that vimentin expression is characterized by a focal, variably sized, green signal near the nucleus and surrounding Golgi apparatus, which did not expand up to the cell membrane of the round‐shaped cells (F, insert). Each arrow represents the same cells from the z‐stack picture to the 3D section view. 3D reconstruction of DH82Ond pi double immunolabelling for vimentin/WGA was obtained by 115 z‐stack frames (0.13 µm steps)

Non‐infected (mean = 98%; median = 99%; range: 97%‐99%) and persistently CDV‐infected (mean = 98%; median = 98%; range: 97%‐99%) DH82 cells (Figure 4F‐H) lacked a significant difference in the percentage of vimentin‐expressing cells (*P* = .7137). When evaluated on the basis of the different cell phenotypes, the number of round cells expressing vimentin was significantly (*P* = .0076) higher among DH82Ond pi cells compared to non‐infected controls. In contrast, non‐infected DH82 cells revealed a significantly higher number of vimentin‐positive spindle‐shaped cells (*P* = .0016) compared to DH82Ond pi cells (Figure 4)I). A significantly (*P* = .0132) higher percentage of non‐infected DH82 cells displayed a diffuse cytoplasmic vimentin expression compared to DH82Ond pi cells (Figure 4J). In contrast, a significantly (*P* = .0035) higher percentage of DH82Ond pi cells showed a focal cytoplasmic expression of vimentin compared to non‐infected controls. In order to confirm this finding, a 3D reconstruction from a double‐labelling immunofluorescence combining vimentin and WGA was obtained (Figure 5). The 3D reconstructions revealed that non‐infected DH82 cells with a round morphology displayed a variably sized and shaped focal immunopositive area located near the nucleus and the Golgi apparatus, which did not expand to the cell membrane. In contrast, spindle‐shaped cells exhibited a diffuse cytoplasmic staining that extended until immediately below the cell membrane (Figure 5A‐C). Similar to non‐infected cells with a round morphology, DH82Ond pi cells often displayed a focal immunoreactivity not expanding to the cell membrane (Figure 5D‐F). Additional single‐labelling immunofluorescence pictures displaying the intracellular distribution of N‐cadherin and vimentin in non‐infected and persistently CDV‐infected DH82 cells at different confluences are available as Figure S8.

### 3.4 Molecular expression of mesenchymal and epithelial markers in DH82Ond pi is suggestive of MET

Selection of gene symbols and proteins associated with EMT/MET, invasion and angiogenesis resulted in a manually generated list of 84 canine gene symbols (Table S1). Among the selected gene symbols, 38 were differentially expressed between DH82Ond pi cells and non‐infected controls. When specifically analysed according to the functional grouping, 18 genes related to EMT/MET were down‐regulated while 11 were up‐regulated (Table 2). Among the epithelial markers previously investigated, only cytokeratin 8 gene symbol was up‐regulated in DH82Ond pi cells. Interestingly, among the up‐regulated genes also TWIST1 was included, one of the most important transcription factors involved in the activation of the EMT/MET process.^17^, ^29^, ^30^ Additionally, the gene symbol of myoferlin (MYOF), another protein associated with the expression of epithelial and mesenchymal markers,^31^, ^32^ was down‐regulated. This observation might further correlate with the down‐regulation of fibronectin‐1 (FN1).

**Table 2:** Manually generated list of canine gene symbols associated with epithelial to mesenchymal/mesenchymal to epithelial transition (EMT/MET), invasion, and angiogenesis, differentially expressed in non‐infected and persistently canine distemper virus‐infected DH82 cells

Canine gene symbol	Fold change	*P*‐value	Gene name	Functional group	References
TUBA4A	−196.54	<0.001	Tubulin, alpha 4a	Invasion and angiogenesis	43
LAMA3	−52	<0.001	Laminin α3	Invasion and angiogenesis	44
WLS	−51.94	<0.001	Wntless	EMT/MET	45, 46
ITGA7	−30.73	<0.001	Integrin α7	Invasion and angiogenesis	44
CXCR4	−13.68	<0.001	Chemokine (C‐X‐C motif) receptor 4	Invasion and angiogenesis	44
LTBP1	−10.72	<0.001	Latent transforming growth factor beta‐binding protein 1	EMT/MET	48, 49
CLTCL1	−8.998	<0.001	Clathrin, heavy chain‐like1	EMT/MET	50
IGF2R	−7.35	<0.001	Insulin‐like growth factor 2 receptor	Invasion and angiogenesis; EMT/MET	17, 44, 51
IGFBP7	−6.16	<0.001	Insulin‐like growth factor binding protein 7	Invasion and angiogenesis; EMT/MET	17, 44, 51
BHLHE41	−4.32	<0.001	Basic helix loop helix e41	EMT/MET	52
RAB6B	−3.57	<0.001	RAB6B, member RAS oncogene family	EMT/MET	53
CAV1	−3.381	<0.001	Caveolin 1	EMT/MET	54
RASA1	−3.3	<0.001	RAS p21 protein activator (GTPase activating protein) 1	EMT/MET	53
TGFB2	−3.2	<0.001	Transforming growth factor, beta 2	EMT/MET	55
CAV2	−3.03	<0.001	Caveolin 2	EMT/MET	56
FN1	−2.7	<0.001	Fibronectin 1	EMT/MET	17
ITGA6	−2.6	<0.001	Integrin α6	Invasion and angiogenesis	44
ITGB1	−2.54	<0.001	Integrin, beta 1	Invasion and angiogenesis; EMT/MET	44
RAB13	−2.38	<0.001	RAB13, member RAS oncogene family	EMT/MET	53
LEF1	−2.29	<0.001	Lymphoid enhancer‐binding factor 1	EMT/MET	45, 46
FZD2	−1.99	<0.001	Frizzled family receptor 2	EMT/MET	45, 46
MYOF	−1.9	<0.001	Myoferlin	Invasion and angiogenesis; EMT/MET	31, 32, 36
FGF2	−1.842	0.003	Fibroblast growth factor 2 (basic)	Invasion and angiogenesis	44
LRP1	−1,721	0.002	low‐density lipoprotein receptor‐related protein 1	Invasion and angiogenesis	44
AMFR	−1.528	0.0422	autocrine motility factor receptor, E3 ubiquitin protein ligase	Invasion and angiogenesis	44
ILK	−1.51	0.0257	integrin‐linked kinase	Invasion and angiogenesis	44
PDGFRL	1.554	0.004	Platelet‐derived growth factor receptor‐like	EMT/MET	57
TGFBR1	1.7	<0.001	Transforming growth factor, beta receptor 1	EMT/MET	17
CSNK1G1	1.844	<0.001	Casein kinase 1, gamma 1	EMT/MET	45, 46
GSK3B	2.44	0.0781	Glycogen synthase kinase 3 beta	EMT/MET	45, 46
CD44	2.65	<0.001	CD44 molecule (Indian blood group)	EMT/MET	17
SENP7	2.66	<0.001	SUMO/sentrin specific peptidase 7	EMT/MET	30
TWIST1	3.03	<0.001	Twist1	EMT/MET	13, 17
CTNND1	3.06	<0.001	catenin (cadherin‐associated protein), delta 1	EMT/MET	17
TGFBI	6.46	<0.001	Transforming growth factor, beta‐induced	EMT/MET	17
KRT8	18.38	<0.001	Keratin 8	EMT/MET	17
CDH2	77.29	<0.001	Cadherin 2, type 1, N‐cadherin (neuronal)	EMT/MET	13, 17

Note

Microarray data were obtained from a previously published dataset^13^, ^14^ and were filtered according to a combination of the fold change (FC ≥ 1.5 or ≤−1.5) and the level of significance (*P* ≤ .05). Down‐regulated genes are highlighted in green, while up‐regulated genes are labelled in red.

Summarized, a higher percentage of DH82Ond pi cells expressed typical epithelial markers compared to non‐infected controls. Additionally, the expression of typical mesenchymal markers was maintained in both non‐infected and persistently CDV‐infected DH82 cells. Taken together, these results are indicative of a virus‐induced mesenchymal to epithelial transition process in canine histiocytic sarcoma cells. Considered that the MET process is known to reduce the invasiveness and angiogenesis in many types of sarcomas,^17^ further investigations regarding gene symbols associated with invasion and angiogenesis within the microarray dataset, and functional analyses of cell motility and invasiveness were performed.

### 3.5 MET in DH82Ond pi cells is associated with a decreased cell motility and invasiveness

Analysis of the aforementioned microarray dataset revealed that among 19 selected genes classified within the functional group ‘invasion and angiogenesis’, all 13 differentially expressed gene symbols between DH82Ond pi and non‐infected controls were down‐regulated (Table 2). This observation is suggestive of a reduced activation of intracellular pathways associated with tumour invasion, and/or angiogenesis which might be the consequence of the activation of a MET process.

**Figure 6:**
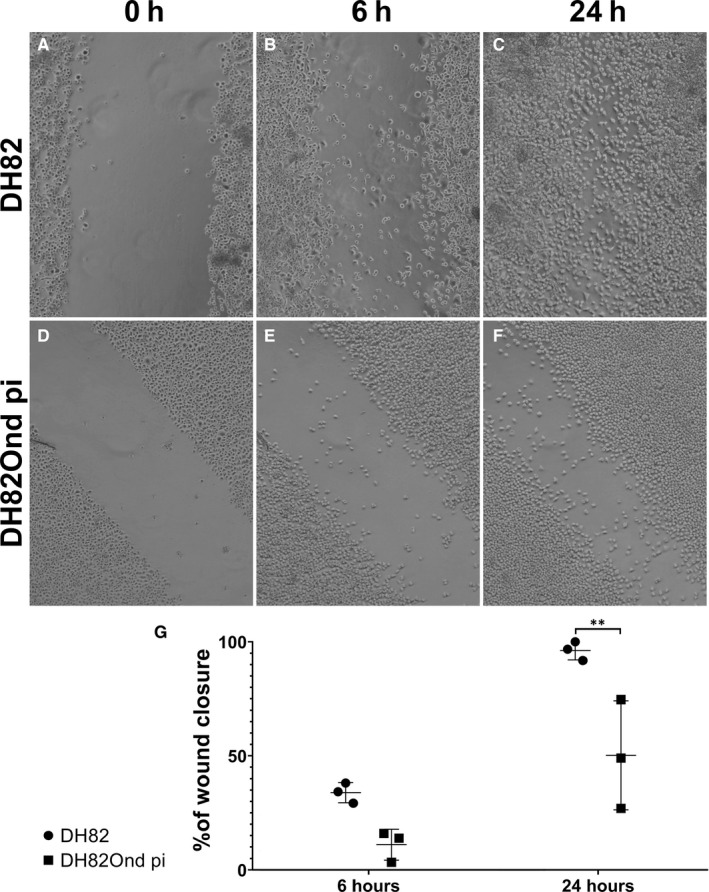
Representative pictures of the scratch assay at time point 0 h (A, D), 6 h (B, E) and 24 h (C, F) in non‐infected DH82 (A, B, C) and persistently CDV‐infected DH82 cells (D, E, F). The percentage of closure of the scratch in non‐infected DH82 (B) and DH82Ond pi (E) was not significantly different after 6 h as shown in (G). The wound closure at 24 h after the scratch was significantly higher in non‐infected DH82 (C) compared to persistently CDV‐infected DH82 cells (F) as shown in (G)

To further investigate the functional relevance of the molecular and protein expression findings, a scratch and an invasion assay were performed with persistently CDV‐Ond infected DH82 cells and non‐infected controls. To assess cell motility, the percentage of wound closure was measured at 6 and 24 hours after scratching of a monolayer of each cell population (Figure 6). After 6 hours, no significant differences (*P* = .1161) were found in the percentage of wound closure in non‐infected DH82 compared to DH82Ond pi cells.

**Figure 7:**
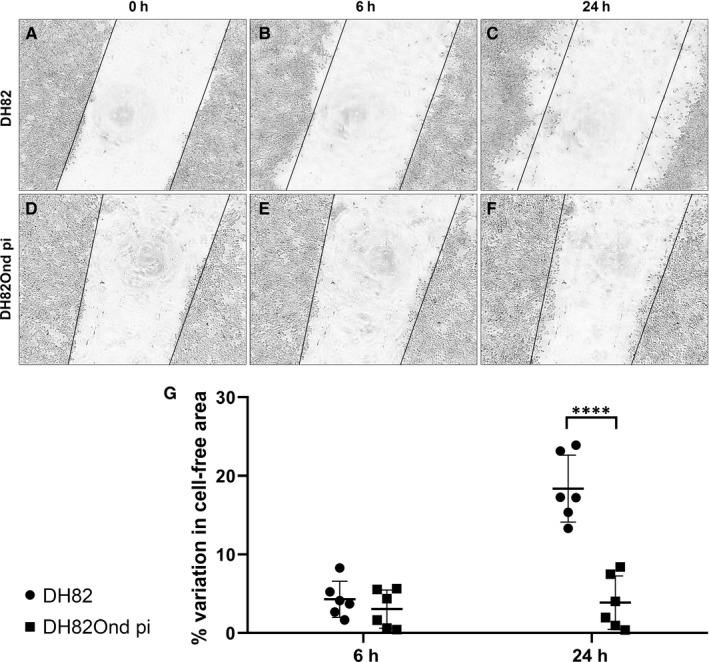
Representative pictures of the invasion assay at time point 0 h (A, D), 6 h (B, E) and 24 h (C, F) in non‐infected DH82 (A, B, C) and persistently CDV‐infected DH82 cells (D, E, F). Non‐infected DH82 cells showed a tendency to move towards the periphery of the well forming cell aggregates that projected upward through the Matrigel matrix (B, C), while DH82Ond pi cells retained a monolayered growth pattern, associated with a weak tendency to move towards the scratch. The percentage of cell‐free area in non‐infected DH82 (B) and DH82Ond pi (E) was not significantly different after 6 h as shown in (G). On the contrary, after 24 h a significantly higher percentage of cell‐free area was observed in non‐infected controls (C) compared to persistently CDV‐infected DH82 cells (F) as displayed in (G), suggesting a reduced invasive potential for the latter

To assess cell invasiveness, the percentage of cell‐free area was evaluated at 6, 24 and 144 hours after scratching of a monolayer of each cell population followed by covering with Matrigel matrix (Figure 7). Already after 6 hours, non‐infected DH82 cells showed a tendency to move towards the periphery of the well forming cellular aggregates. On the contrary, DH82Ond pi cells maintained an arrangement in a monolayer, associated with a weak tendency to move towards the scratch. Nonetheless, no significant differences (*P* = .7591) were found in the percentage of cell‐free area of non‐infected compared to DH82Ond pi cells at this time point. After 24 hours, non‐infected DH82 cells were mostly arranged in large peripheral aggregates projecting upward through the Matrigel matrix. This observation was associated with a significantly (*P* < .0001) higher percentage of cell‐free area compared to DH82Ond pi cells, which kept their monolayer arrangement associated with a mild tendency to invade the scratch. After 144 hours, both non‐infected DH82 and DH82Ond pi cells showed clear signs of cellular necrosis characterized by cell shrinkage and by the accumulation of abundant cellular debris within each well. Therefore, the evaluation of the cell‐free area at this time point was excluded from the statistical analysis. These data indicated that non‐infected DH82 cells have a higher migration and invasion potential as demonstrated by a faster wound closure and by the ability to migrate through the Matrigel to form aggregates. Summarized, it could be assumed that a MET induced by the CDV infection contributes to a decreased cell motility of DH82 cells in vitro.

## 4 DISCUSSION

The aim of the current study was to investigate the impact of CDV‐Ond infection on DH82 cells, a histiocytic sarcoma cell line, on the induction of mesenchymal to epithelial transition, and if this process resulted in a decreased motility of the neoplastic cells.

In the present study, as detected with immunofluorescence and confirmed by immunoblotting, E‐cadherin was significantly over‐expressed in DH82Ond pi cells compared to non‐infected controls. This observation was further substantiated by an increased protein expression of cytokeratin 8 in DH82Ond pi compared to non‐infected DH82 cells. Additionally, the expression of β‐catenin was also increased in persistently CDV‐infected DH82 cells, although no significant differences were noted. Taken together, these results are indicative of the occurrence of MET in DH82 cells that might be the direct consequence of the infection with CDV‐Ond. In the literature, the expression of epithelial markers in several types of sarcomas has been correlated with the development of MET.^17^, ^18^, ^19^, ^33^, ^34^, ^35^ This event has been associated with a better clinical outcome,^16^, ^30^, ^31^ suggesting an emerging role of MET in sarcomas as a potential biological and positive prognostic factor related with reduced invasiveness and metastatic rate.^17^ In this context, the CDV‐driven MET process observed in the current study could represent a promising hint for the use of CDV‐Ond as an oncolytic virus, in addition to the already reported antitumoral effects associated with the viral infection such as the alteration of MMP expression, cortactin distribution, and tumour‐associated vascularization and angiogenesis.^12^, ^13^, ^14^

Despite the promising consequences of MET, the underlying mechanisms have been only marginally detailed so far.^17^ Nonetheless, additional molecules other than epithelial and mesenchymal markers have been correlated with MET, such as myoferlin.^31^ In the current study, DH82Ond pi cells showed a significant down‐regulation of the myoferlin gene (MYOF) compared to non‐infected DH82 cells. A depletion of this molecule in breast cancer has been associated with a reversion of the EMT process, affecting tumour invasiveness.^31^, ^32^, ^36^ Specifically, the associated increase of expression of E‐cadherin and reduced levels of fibronectin and vimentin highlighted that MYOF plays an important role in EMT/MET phenomenon.^31^ These data are partially in line with the results obtained in the current study, in which the down‐regulation of MYOF was associated simultaneously with a down‐regulation of the fibronectin gene (FN1) and with an increased protein expression of E‐cadherin. TWIST represents another important regulator of the MET process,^17^, ^29^ which directly interacts with the expression of genes associated with the epithelial and mesenchymal phenotype. TWIST can down‐regulate the E‐cadherin and activate the transcription of genes such as N‐cadherin and vimentin, which are associated with a mesenchymal phenotype.^29^ Interestingly, the mRNA data from the current study displayed an increased expression of TWIST together with an up‐regulation of N‐cadherin. However, at the protein level, DH82Ond pi cells displayed an increased E‐cadherin protein expression while a high expression of N‐cadherin was present only at the mRNA level. This might be attributed to an incomplete MET status, in which DH82Ond pi cells might be still in a hybrid transient phase with a so‐called ‘metastable phenotype’.^21^ Similarly, the expression of both E‐cadherin and N‐cadherin at the protein level has been reported for circulating breast cancer cells, expressing mixed epithelial and mesenchymal characteristics in a hybrid state.^37^ Additionally, the discrepancies observed in the current study between the mRNA amount and the expression of the corresponding protein might be related to the intervention of miRNAs, which play a well‐known role in the EMT and MET process,^15^ being able to directly influence the expression of E‐cadherin and N‐cadherin. However, the role of miRNAs in the MET process in DH82Ond pi cells should be taken in consideration for future investigations.

Compared to non‐infected controls, DH82Ond pi cells over‐expressing E‐cadherin showed more frequently an unexpected localization of this protein in a focal area of the cytoplasm around the nucleus and the Golgi apparatus, despite not reaching statistical significance. Interestingly, E‐cadherin cytoplasmic internalization and other post‐translational modifications of the EMT/MET effectors might be involved in the uncommon expression of this epithelial marker at the perinuclear level.^29^

In the current study, cytokeratin 8 also displayed a different intracellular localization depending on the cellular infection status. Notably, non‐infected DH82 cells were characterized by a significantly more frequent cytoplasmic expression of cytokeratin 8, which was focally arranged around the nucleus and the Golgi apparatus, whereas DH82Ond pi cells showed a pronounced expression of variably sized aggregates of this protein within the cytoplasm and intermingled with the cell membrane. Interestingly, the MET process seems to be associated with a decreased cell motility in DH82Ond pi cells. Indeed, as already reported, a knockdown of cytokeratin 8 and 18 in neoplastic epithelial cells was associated with a significantly increased cancer cell motility and invasiveness.^38^ In addition, cytoplasmic expression of cytokeratin 8 is linked to a general inhibition of the migratory potential, while a perinuclear localization is related to an increased tumour cell motility.^39^ Similar results were obtained in the present study, which revealed an up‐regulation of cytokeratin 8 within DH82Ond pi cells compared to non‐infected controls The observation of cytokeratin 8 mainly in a ‘membranous to cytoplasmic’ localization in DH82Ond pi cells might be one factor leading to the reduced cell motility observed in the scratch and in the invasion assay. In contrast, an increased perinuclear expression of cytokeratin 8 in non‐infected DH82 cells was associated with an increased cell motility in both functional assays applied.

Furthermore, intermediate filaments and specifically cytokeratins are involved in cell adhesion, localization of the organelles, and changing of cellular shape.^40^ This might be correlated with the more homogeneous round cellular shape of DH82Ond pi cultures compared to non‐infected controls, with the latter characterized by a more pleomorphic phenotype. The hypothesis of a reduced cell motility due to a rearrangement of intermediate filaments during the MET process might be further supported by the fact that a reduced expression of vimentin is also associated with a reduced cell motility during MET.^41^ In the present study, a similar number of cells expressed vimentin regardless of the infection status. However, DH82Ond pi and non‐infected DH82 cells showed a higher number of positive round and spindle cells, respectively. This finding was mirrored by the fact that DH82Ond pi and non‐infected DH82 cells showed a more frequent focal and diffuse cytoplasmic distribution of vimentin, respectively, which was confirmed by 3D reconstructions. Taken together, these results suggest that the different intracellular distribution of vimentin between DH82Ond pi and non‐infected controls might be correlated to the predominant cellular phenotype among each cell population, rather than to the infection status. Nevertheless, the predominant cellular phenotype among each cell population seems to be dependent on the infection status, thus suggesting an indirect role of the virus in the intracellular redistribution of vimentin. Interestingly, a spontaneous CDV infection of canine brain cells has also been reported to modify cytoskeletal proteins such as vimentin and glial fibrillary acid protein (GFAP) in vivo.^25^ Considered that the literature highlighted the fundamental influence of the cell shape on motility,^40^, ^42^ virus‐induced morphological and structural (ie intermediate filament rearrangement) modifications might be the cause of the observed alterations in cell motility and invasiveness.

In conclusion, the results of the present study are suggestive of a MET process in DH82 cells driven by CDV infection, as shown by an increased expression of epithelial markers in DH82Ond pi cells. Additionally, CDV‐driven MET seems to affect invasiveness and cell motility in vitro, most likely based on a rearrangement of cytoskeletal intermediate filaments. Nevertheless, future studies are warranted to detail the impact of the different factors involved in MET processes in DH82Ond pi cells.

## CONFLICT OF INTEREST

The authors declare no potential conflicts of interest.

## AUTHOR CONTRIBUTIONS

**Federico Armando:** Conceptualization (equal); data curation (equal); formal analysis (equal); investigation (equal); methodology (equal); validation (equal); visualization (equal); writing‐original draft (equal); writing‐review and editing (equal). **Matteo Gambini:** Data curation (equal); formal analysis (equal); investigation (equal); methodology (equal); validation (equal); visualization (equal); writing‐original draft (equal); writing‐review and editing (equal). **Attilio Corradi:** Supervision (equal); writing‐review and editing (equal). **Kathrin Becker:** Investigation (equal); methodology (equal); validation (equal); writing‐review and editing (equal). **Katarzyna Marek:** Formal analysis (equal); investigation (equal); methodology (equal); validation (equal); writing‐review and editing (equal). **Vanessa Maria Pfankuche:** Investigation (equal); validation (equal); writing‐review and editing (equal). **Ahmed Elmonastir Mergani:** Formal analysis (equal); investigation (equal); methodology (equal); validation (equal); visualization (equal); writing‐review and editing (equal). **Graham Brogden:** Formal analysis (equal); investigation (equal); methodology (equal); validation (equal); visualization (equal); writing‐review and editing (equal). **Nicole de Buhr:** Formal analysis (equal); investigation (equal); validation (equal); visualization (equal); writing‐review and editing (equal). **Maren von Köckritz‐Blickwede:** Investigation (equal); project administration (equal); resources (equal); supervision (equal); validation (equal); writing‐review and editing (equal). **Hassan Y. Naim:** Resources (equal); supervision (equal); writing‐review and editing (equal). **Wolfgang Baumgärtner:** Conceptualization (equal); project administration (equal); resources (equal); supervision (equal); validation (equal); writing‐original draft (equal); writing‐review and editing (equal). **Christina Puff:** Conceptualization (equal); data curation (equal); formal analysis (equal); project administration (equal); supervision (equal); validation (equal); visualization (equal); writing‐original draft (equal); writing‐review and editing (equal).

## Supporting information

Figure S1

Figure S2

Figure S3

Figure S4

Figure S5

Figure S6

Figure S7

Figure S8

Table S1

Table S2

## Data Availability

The data that support the findings of this study are included within the main document or supporting supplementary material. The remaining data, which are not included in the main document or supplementary material, are available from the corresponding author upon reasonable request.
