# Supplementary figures and images for: Mesenchymal to epithelial transition driven by canine distemper virus infection of canine histiocytic sarcoma cells contributes to a reduced cell motility in vitro
Source: J Cell Mol Med. 2020 Jul 6;24(16):9332–48. doi: 10.1111/jcmm.15585 (PMC7417708; doi:10.1111/jcmm.15585)

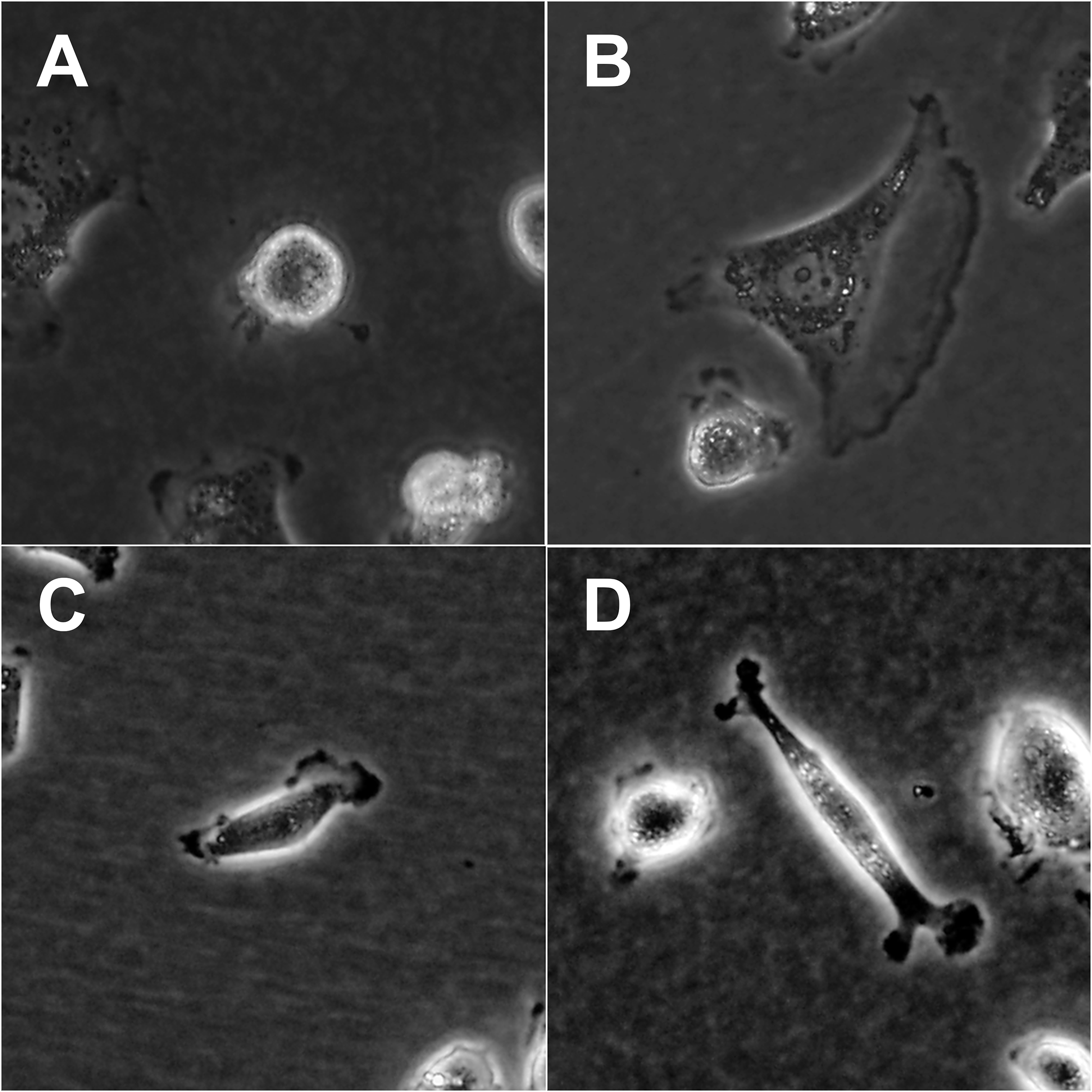

Supplement: Supplementary file 1 — Figure S1 [file JCMM-24-9332-s001.tif]

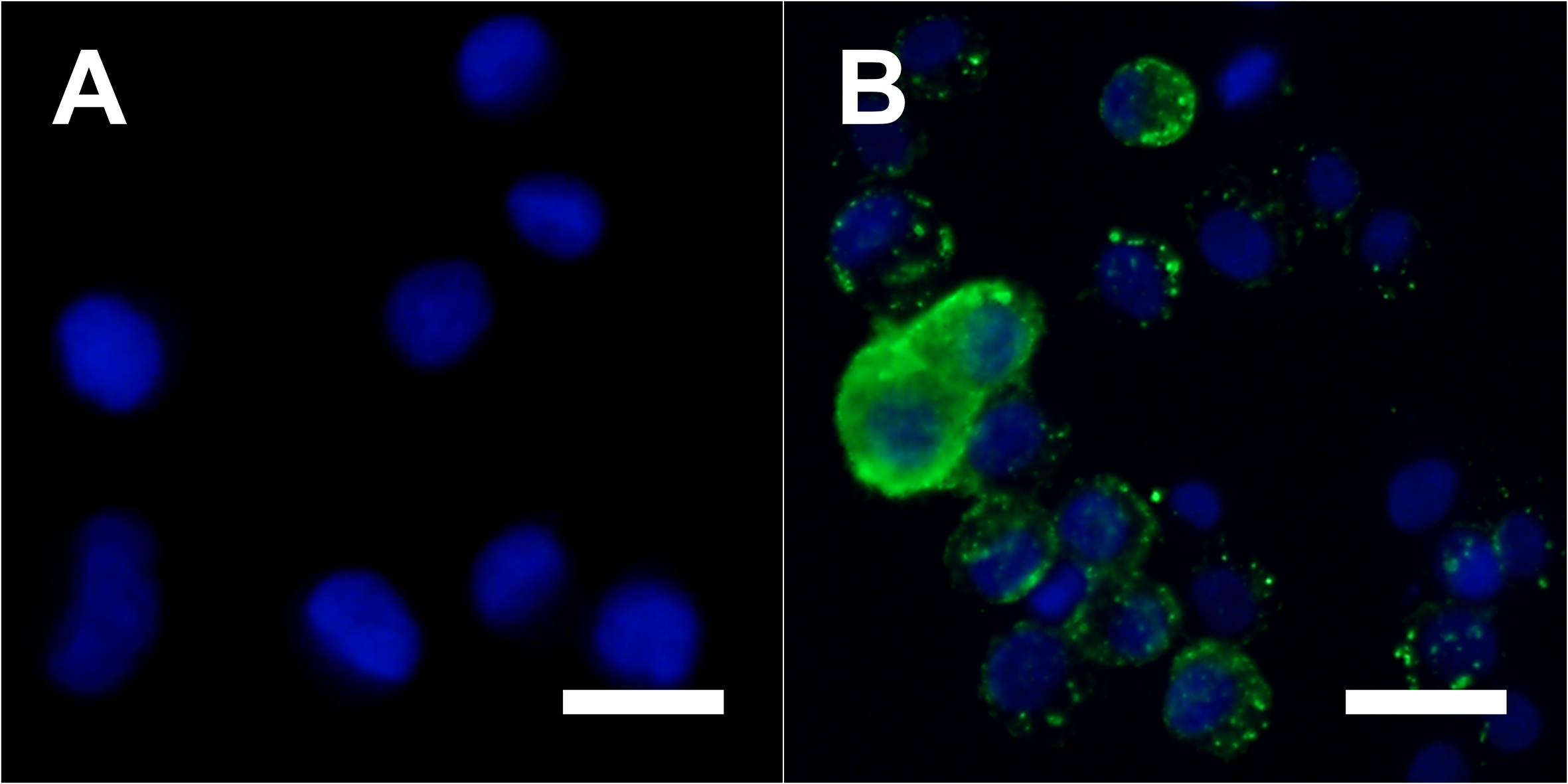

Supplement: Supplementary file 2 — Figure S2 [file JCMM-24-9332-s002.tif]

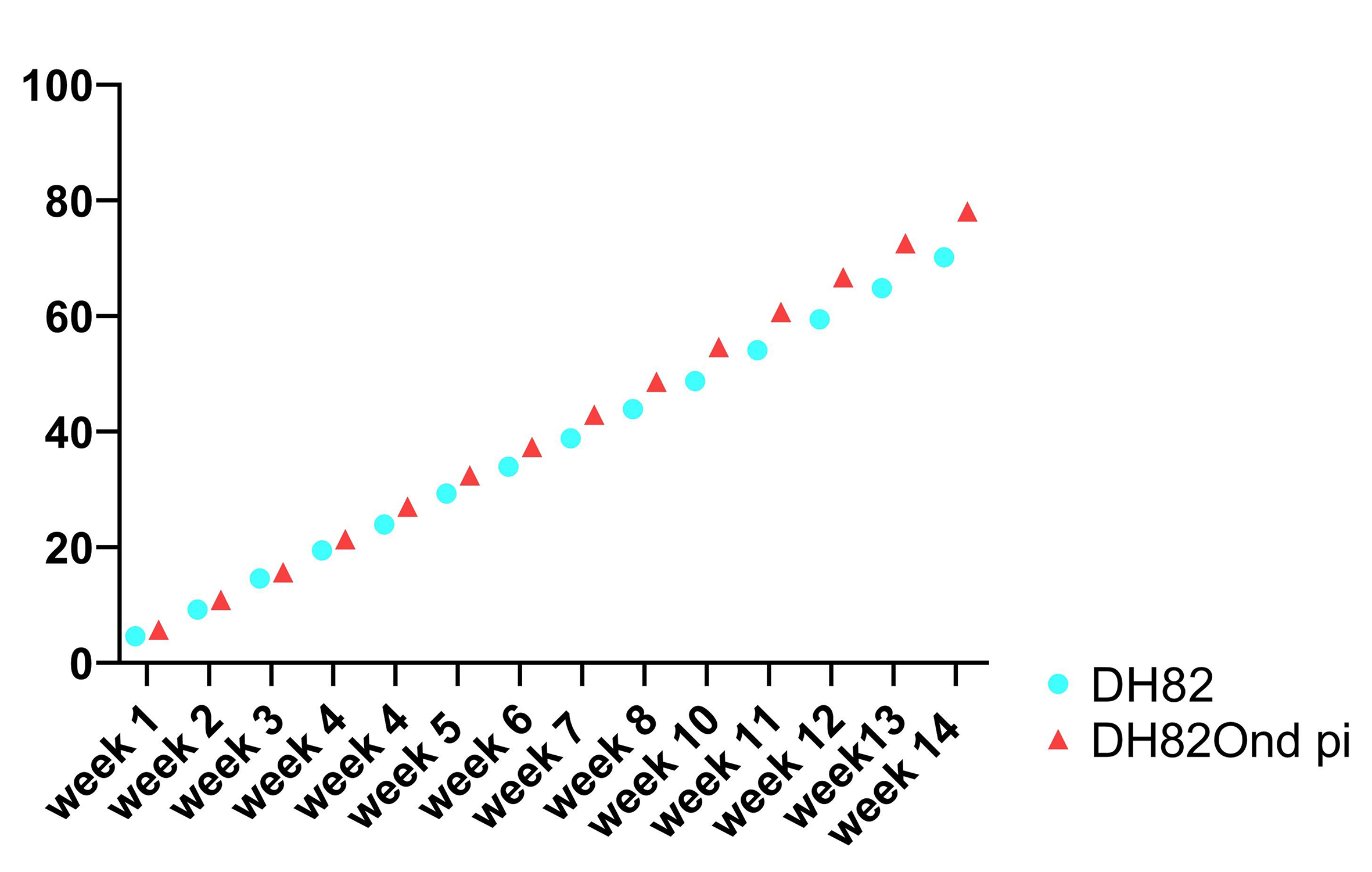

Supplement: Supplementary file 3 — Figure S3 [file JCMM-24-9332-s003.tif]

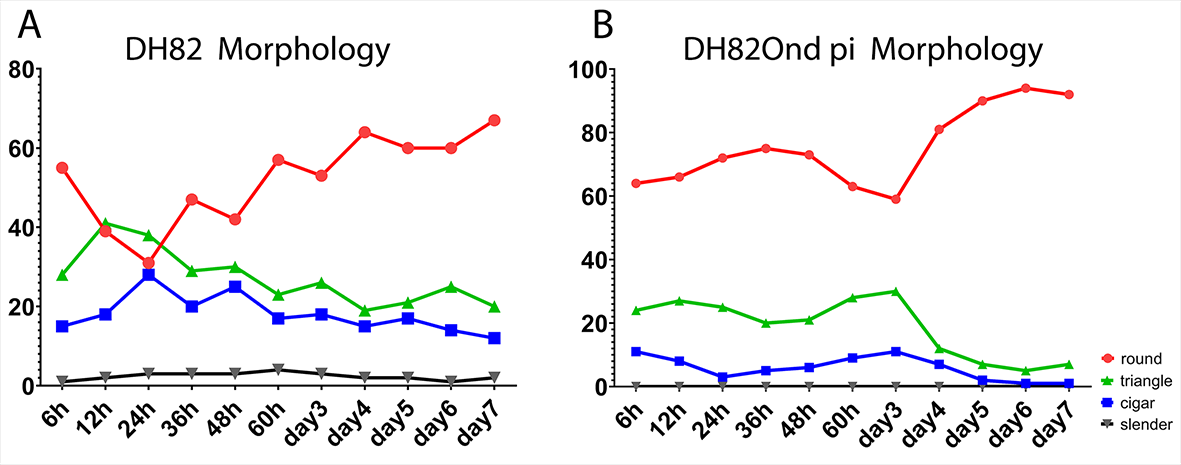

Supplement: Supplementary file 4 — Figure S4 [file JCMM-24-9332-s004.tif]

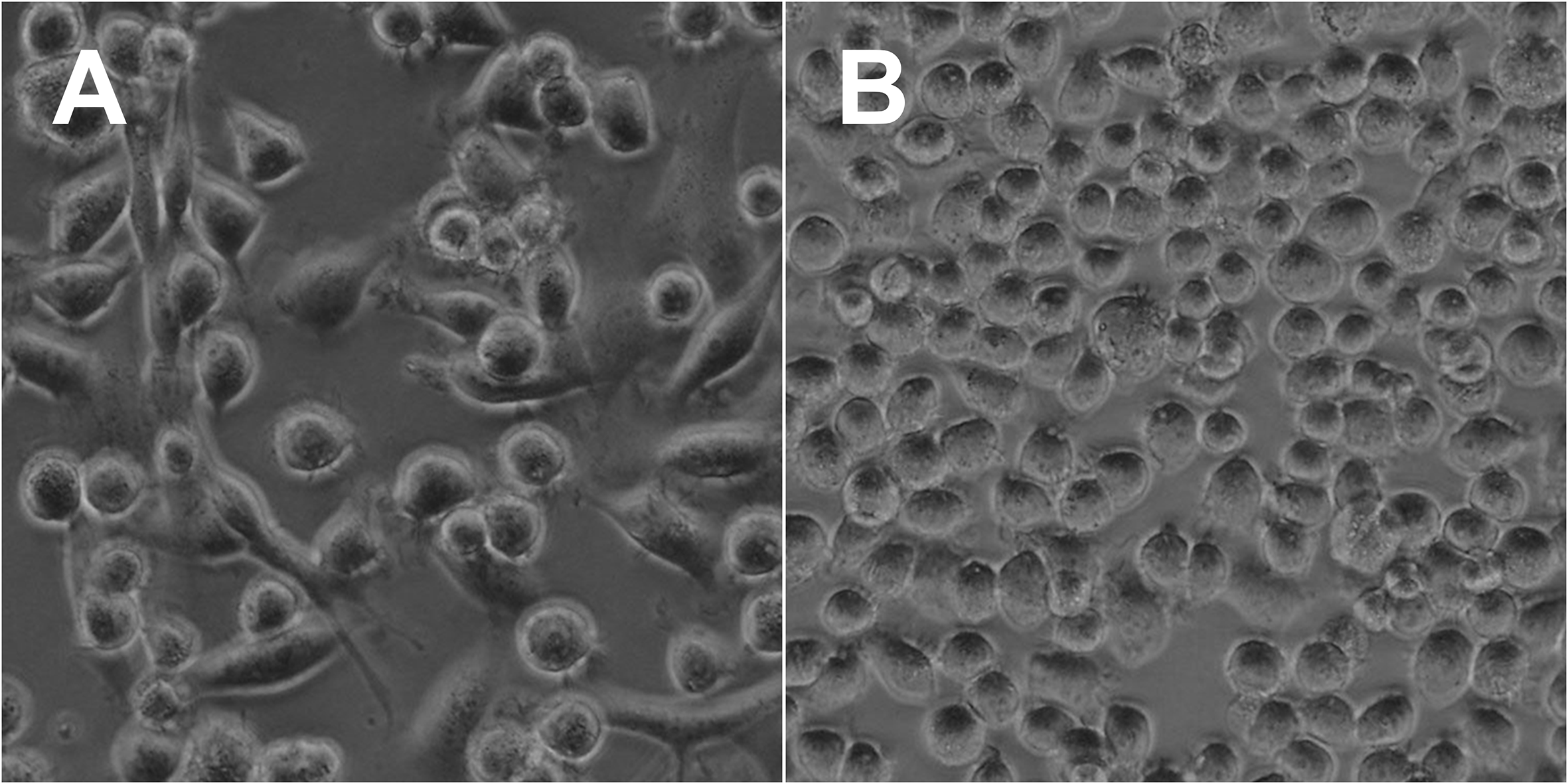

Supplement: Supplementary file 5 — Figure S5 [file JCMM-24-9332-s005.tif]

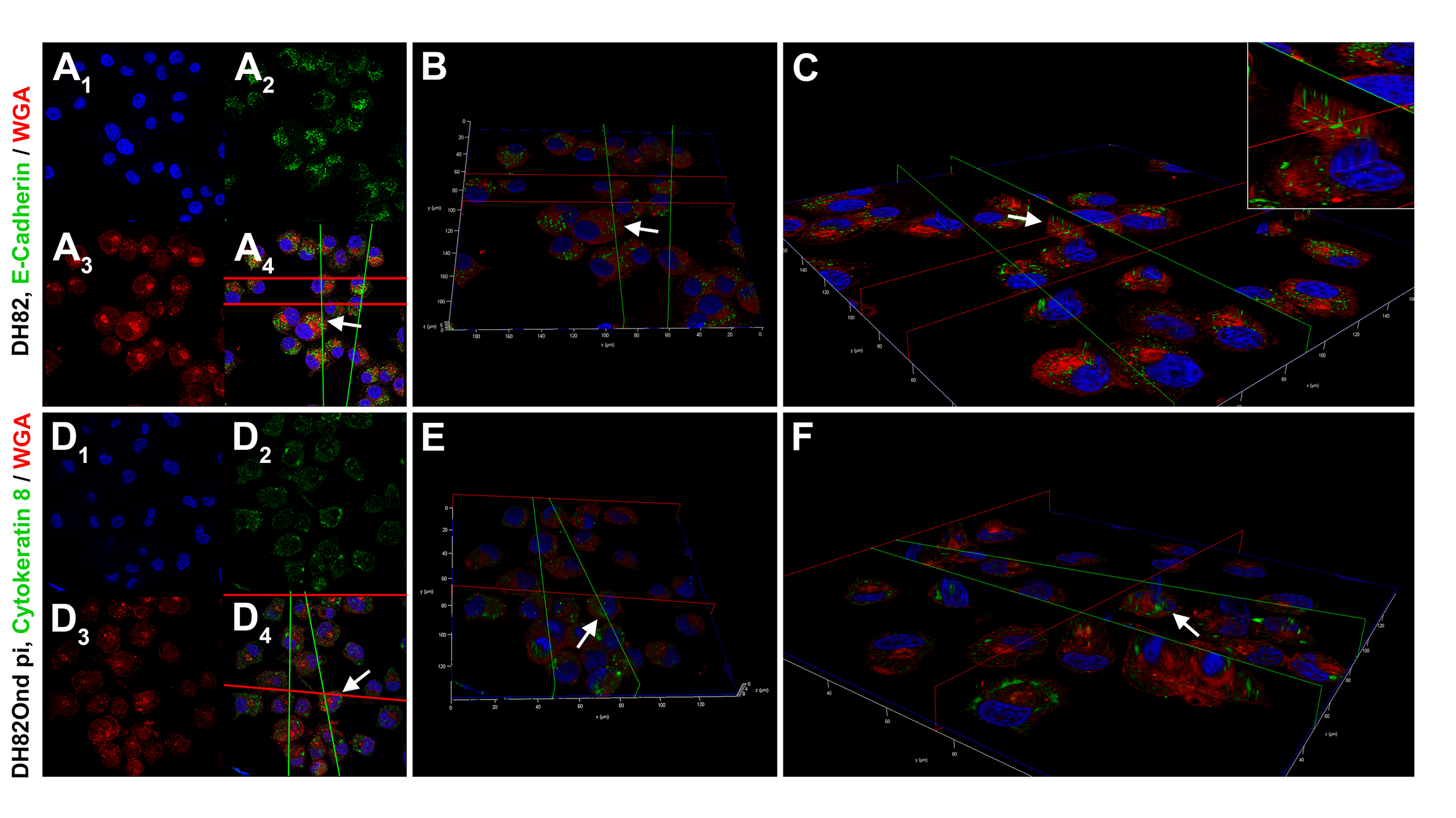

Supplement: Supplementary file 6 — Figure S6 [file JCMM-24-9332-s006.tif]

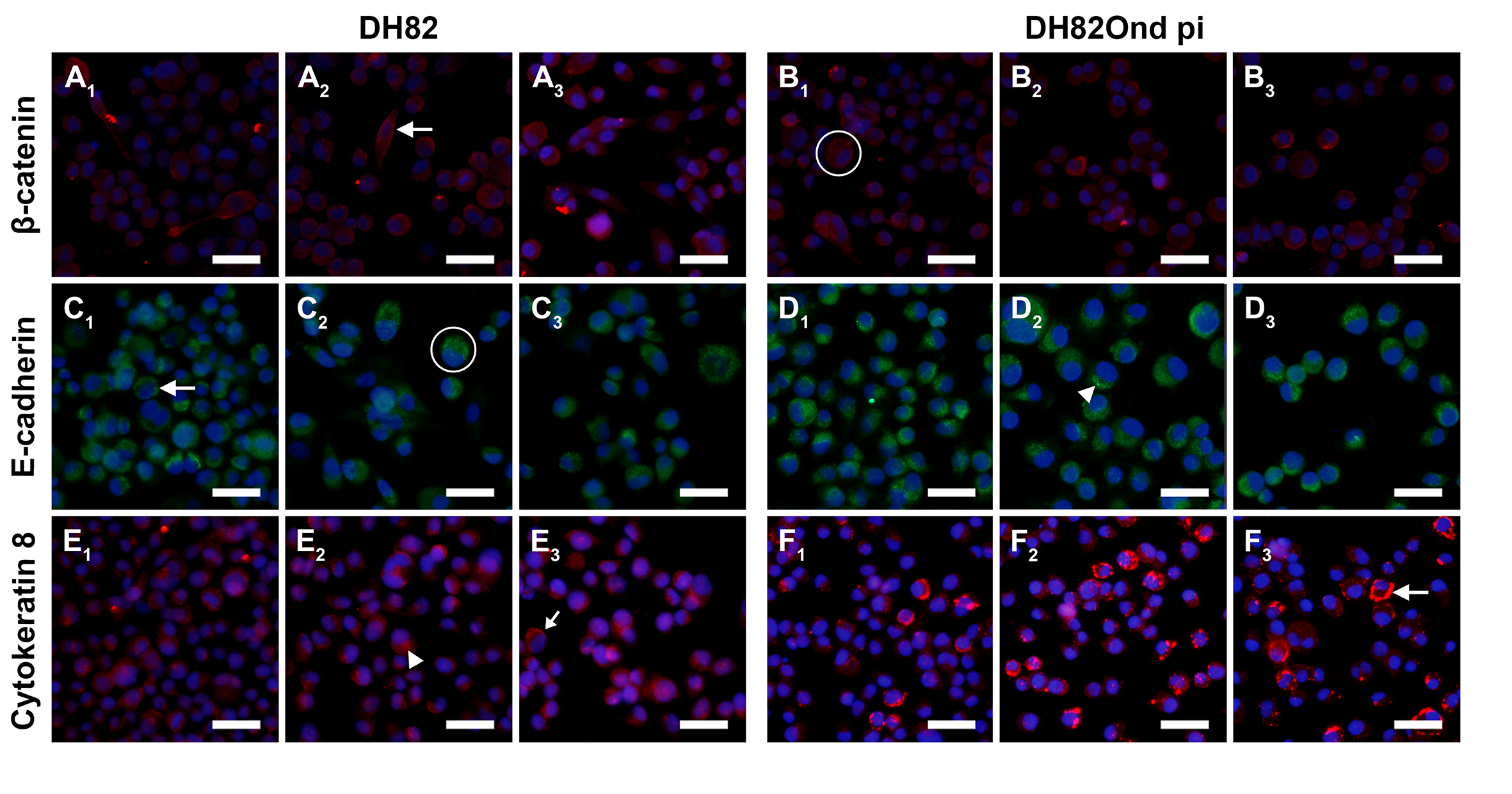

Supplement: Supplementary file 7 — Figure S7 [file JCMM-24-9332-s007.tif]

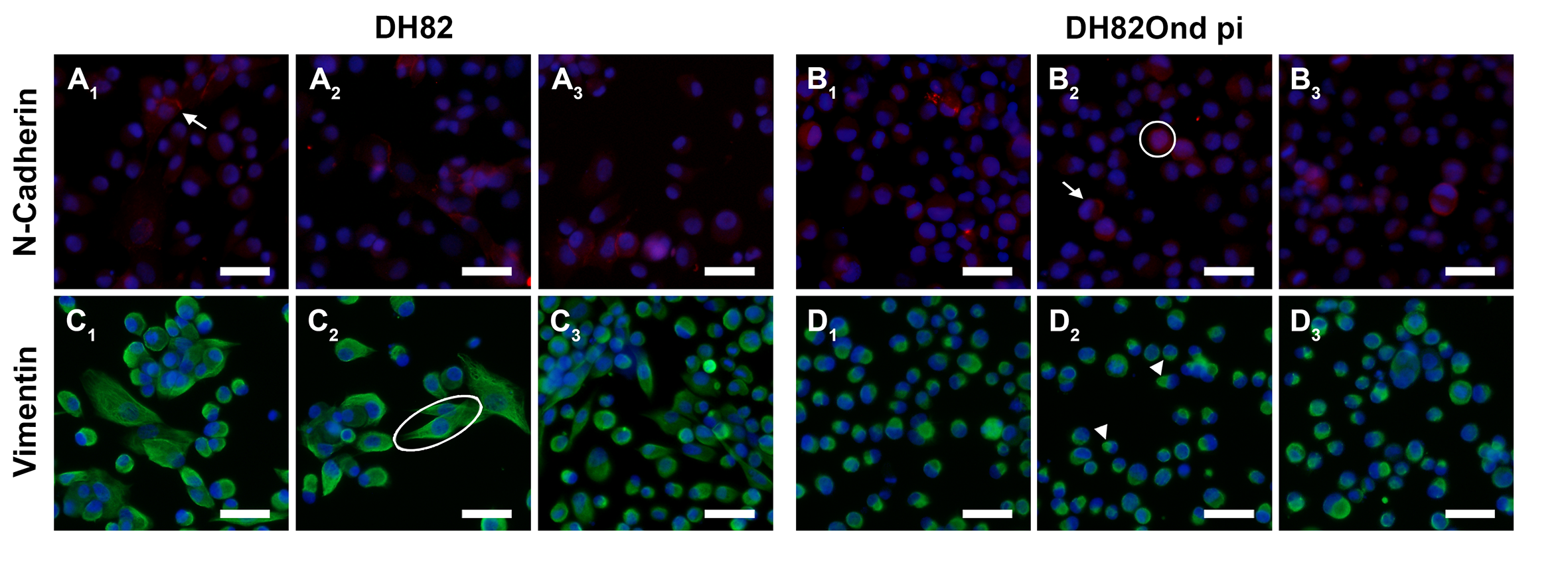

Supplement: Supplementary file 8 — Figure S8 [file JCMM-24-9332-s008.tif]
