# Supplementary material for: Mesenchymal to epithelial transition driven by canine distemper virus infection of canine histiocytic sarcoma cells contributes to a reduced cell motility in vitro
Source: J Cell Mol Med. 2020 Jul 6;24(16):9332–48. doi: 10.1111/jcmm.15585 (PMC7417708; doi:10.1111/jcmm.15585)
Supplement: Supplementary file 10 — Table S2 [file JCMM-24-9332-s010.docx]

**Mesenchymal to epithelial transition driven by canine distemper virus infection of canine histiocytic sarcoma cells contributes to a reduced cell motility *in vitro***

F. Armando^1,2¶^, M. Gambini^1,3¶^ A. Corradi^1,2^, K. Becker^1^, K. Marek^1^, V. M. Pfankuche^1^, A. E. Mergani^4^, G. Brogden^4,5^, N. de Buhr^4^, M. von Köckritz-Blickwede^4,6^, H. Y. Naim^4^, W. Baumgärtner^1^*, C. Puff^1^

**Supplementary material:**

**This file includes:**

Additional details for materials and methods and corresponding results

Figure legends: 1 to 8

Tables: 2

Preliminary immunohistochemical testing of epithelial and mesenchymal markers on DH82 cell pellets

Three formalin-fixed paraffin embedded (FFPE) cell pellets of non-infected DH82 cells and 3 of DH82Ond pi cells were analyzed. Briefly, cells were scraped and centrifuged at 250 g for 10 minutes at 4°C. After removing of the supernatant, cells were washed in phosphate buffer saline (PBS) and centrifuged again. Following a second wash and centrifugation step, the pellet was fixed in 1.5ml of 10% buffered formalin for 8 hours, and processed for routine paraffin embedding.

Three µm thick sections of FFPE cell pellets were analyzed in triplicates. After inhibition of endogenous peroxidases and pretreatment for antigen retrieval if necessary, serum blocking was performed followed by incubation with the primary and the respective biotinylated secondary antibody. The expression of the different markers, listed in Supplementary Table 2, was visualized by applying the ABC-method with 3,3’-diaminobenzidine-tetrahydrochlorid (DAB) as a chromogen followed by Mayer´s hematoxylin for counterstaining. Canine skin (E-cadherin), canine intestine (β-catenin, cytokeratin 8, vimentin) and canine heart (N-cadherin) served as positive controls. For negative controls, the first antibody was replaced with rabbit serum, Balb/c ascitic fluid, or goat serum, respectively, at corresponding protein concentrations.

The initial qualitative immunohistochemical screening of FFPE cell pellets of non-infected and persistently CDV-infected DH82 cells revealed an expression of E-cadherin, β-catenin, cytokeratin 8, vimentin, and N-cadherin in both cell types.

Morphological data

For both DH82 and DH82Ond pi cells, 10 T75flasks each were checked for morphology of the cells at 6h, 12h, 24h, 36h, 48h, 60h, 3 days, 4 days, 5 days, 6 days and 7 days after seeding. At every time point the flasks were observed using a phase contrast microscope (Olympus IX-70, Olympus Optical Co. GmbH, Hamburg, Germany) equipped with an Olympus DP72 camera and Olympus cell sense standard software version 2.3, at a 20x magnification always by the same operator (F.A.). For every flask having a homogeneously distributed cell growth, 5 pictures were taken at the 4 corners of the flask and one in the middle. The pictures were further analyzed and for every field the presence of the 4 most common morphological cell types was recorded. Cells were classified as 1) round cells of different sizes, 2) triangle shaped cells, presenting a cytoplasm modified in a shape resembling a ”kite”, with a prominently oriented cell protrusion, 3) cigar-shaped cells, presenting an increased longitudinal length, with frequently blunted ends, and resembling a thin cylinder, and 4) slender cells, presenting with a very elongated cytoplasm with frequent cytoplasmic projections and a small cytoplasmic width.

Immunofluorescence staining pattern and shape evaluation

After the determination of the percentage of immunopositive cells for each marker, cell shape (spindle, round, or multinucleated giant cells) and intracellular localization (membranous, membranous to cytoplasmic, diffuse cytoplasmic, focal cytoplasmic) were additionally evaluated. Membranous staining pattern was considered for cells with a membranous staining only, membranous to cytoplasmic was considered for cells with both membranous and cytoplasmic staining, diffuse cytoplasmic was considered for a diffuse cytoplasmic staining pattern, and focal cytoplasmic was considered for immunolabelings other than diffuse cytoplasmic that involved only one focal area of the cytoplasm. Cell shapes were considered as: round for any roundish cell of any dimension, multinucleated giant cell for cells with more than 2 nuclei and a total size larger than 3 cells, and spindle for those cells other than round and multinucleated giant cells (e.g. cigar-shaped, slender, and triangle-shaped cells as described for the phase contrast microscopy morphological analysis).

Immunofluorescence double labeling

Non-infected DH82 and DH82Ond pi cells were seeded at a density of 0.03*10^6^ cells/0.33cm^2^ into 96-Well Plates, No. 1.5 Coverslip, 5 mm Glass Diameter, Uncoated (Mat Tek life sciences). All the immunostainings were performed in triplicates with negative controls in duplicates. 3 days after seeding, cells were fixed with 4% paraformaldehyde for 20 minutes at room temperature (RT), followed by serum blocking using PBS–triton (PBST) + 5% normal goat serum + 3% bovine serum albumin (BSA) for 15 minutes at RT. Primary antibodies were diluted in PBST + 3% BSA as follows: cytokeratin 8, 1:200; vimentin, 1:100; and E-cadherin, 1:200; and were incubated at RT for 90 minutes. Afterwards secondary fluorescence conjugated antibodies were diluted 1:200 in PBST + 3% BSA and subsequently incubated for 120 minutes at RT. Goat-anti-mouse (GAM) labeled with Alexa Fluor (AF) 488 (Dianova) was used for vimentin and E-cadherin while goat-anti-rabbit (GAR)-AF 488-conjugated (Invitrogen) was applied for cytokeratin 8. After washing the wells for 5 minutes with PBST, the Alexa Fluor 633-conjugated lectin WGA (Wheat Germ Agglutinin - AF633 conjugated, Invitrogen) was incubated for 120 minutes, followed by washing with bi-distilled water and bisbenzimide (Sigma-Aldrich Chemie GmbH) nuclear counterstaining. For negative controls, the first antibody was replaced with rabbit serum (cytokeratin 8) or Balb/c ascitic fluid (vimentin, E-cadherin) at corresponding protein concentrations.

Immunoblotting

Cell lysates from non-infected and persistently CDV infected DH82 cells were prepared by freezing and thawing in 1 ml NP-40 buffer (50mM Tris-HCl, 150 mM NaCl, 1% NP-40, 5 mM EDTA, 50 µl protease inhibitor cocktail (1.48 µM Antipain dihydrochloride, 0.768 µM Aprotinin 1.46 µM, 10.51 µM Leupeptin, 1.46 µM Pepstatin A in DMSO, 1 mM PMSF, 50 µg/ml Trypsin inhibitor T9128, pH 8.0), all reagents from Sigma-Aldrich, St. Louis, USA). The correct amount of each cell lysate required for the analysis was calculated based on the protein concentration determined applying the Bradford method, as previously described [27]. Samples were analyzed by SDS-PAGE on 8% gels and subsequently transferred to a Polyvinylidene fluoride (PVDF) membrane as described previously [27]. Polyclonal anti-β-catenin (1:500, Sicgen, Coimbra, Portugal) and anti-CK8 (1:2000, Invitrogen, California, USA) antibodies, monoclonal anti-β-actin (1:200, Santa Cruz, Dallas, USA) and anti-E-cadherin (1:200, BD transduction lab, San Jose, CA, USA) antibodies were used as primary antibody. Polyclonal IgG antibody from rabbit serum served as a negative control (2 µg/ml, Sigma-Aldrich, St. Louis, USA). Secondary anti-rabbit, anti-goat or anti-mouse antibodies conjugated to horseradish peroxidase were used (0.2 µg/ml, ThermoScientific, Schwerte, Germany). Protein bands were visualized using SuperSignal™ West Femto maximum sensitivity western blot chemiluminescence substrate (ThermoScientific, Schwerte, Germany) and a ChemiDoc MP Imaging System (Bio-Rad, Hercules, USA).

***Evaluation of pictures of the invasion assay***

Pictures were analyzed with Fiji [<https://imagej.net/Fiji>] to define and calculate the cell-free area of each picture. Specifically, the following macro was apply to each picture:

run("8-bit");

//run("Brightness/Contrast...");

run("Enhance Contrast", "saturated=0.35");

run("Apply LUT");

run("Subtract Background...", "rolling=50 light");

setAutoThreshold("Default");

//run("Threshold...");

setThreshold(0, 194);

//setThreshold(0, 194);

setOption("BlackBackground", false);

run("Convert to Mask");

run("Analyze Particles...", "pixel show=[Overlay Masks] display summarize in_situ");

run("Labels...", "color=white font=9");

Afterwards, the percentage of cell-free area was calculated according to the following formula: 100 – cell-covered area, with the latter expressed as a percentage automatically calculated by Fiji. The percentage variation of the cell-free area was then calculated according to the following formula: (|Area T_0_ – Area T_x_|)/Area T_0_*100, with T_x_ referring to of the time points evaluated.

**Supplementary Table 1**. List of manually-selected gene symbols related to EMT/MET, invasion, and angiogenesis, with corresponding fold change, p-value and functional group. Gene symbols significantly down- or up-regulated are highlighted in green and red, respectively. Complete bibliographic references can be found in the dedicated section within the main manuscript file, numbered as follows: Das et al. 2012 [45], Dong et al. 2019 [31], Dragoi et al. 2014 [56], Govindarajalu et al. 2018 [52], Janda al. 2006 [50], Krock et al. 2011 [44], Lamouille and Derynck 2014 [30], Lee et al. 2018 [43], Li et al. 2012 [44], Mendez, Kojima, and Goldman 2010 [41], Pfankuche, VM et al. 2016 [13], Prunier and Howe 2005 [55], Saito et al. 2006 [35], Sun et al. 2017 [47], Todorovic et al. 2007 [49], Todorovic et al. 2011 [48], Volakis et al. 2014 [36], Wu et al. 2019 [51], Yang et al. 2014 [17], Zhang et al. 2017 [53].

Supplementary Table 1 is submitted as a separate MS Office Excel file

**Supplementary Table 2:** Details of the antibodies used for the immunohistochemical staining preliminarly performed, including primary antibody, host species, clonality, epitope retrieval, blocking serum, dilution of primary antibody and secondary antibody.

| **Primary antibody** | **Host species, clonality** | **Epitope retrieval** | **Serum blocking** | **Dilution** | **Secondary antibody**  **(1:200)** |
| --- | --- | --- | --- | --- | --- |
| Beta catenin (Sicgen) | Goat, polyclonal | Citrate buffer, microwave  (800 W, 20´) | Rabbit serum | 1:3200 | RaG-b |
| Cytokeratin 8 (Invitrogen) | Rabbit, polyclonal | Citrate buffer, microwave  (800 W, 20´) | Goat serum | 1:1000 | GaR-b |
| E-cadherin  (BD transduction lab) | Mouse, monoclonal clone 36/E-Cadherin (RUO) | Citrate buffer, microwave  (800 W, 20´) | Goat serum | 1:100 | GaM-b |
| N-cadherin (Proteintech) | Rabbit, polyclonal | Citrate buffer, microwave  (800 W, 20´) | Goat serum | 1:32000 | GaR-b |
| Vimentin  (Dako) | Mouse, monoclonal clone V9 | n/a | Goat serum | 1:100 | GaM-b |

Legend: GaM-b, goat anti mouse biotinylated; GaR-b, goat anti rabbit biotinylate, immunohistochemistry; n/a, non applied or non applicable; RaG-b, rabbit anti goat biotinylated.

**Suppl. Fig. 1:**

Representative pictures of the 4 different morphological phenotypes. Characteristical round (A), triangle-shaped (B), cigar-shaped (C) and slender cells (D) are shown.

**Suppl. Fig. 2:**

Immunolabeling for canine distemper virus nucleoprotein (CDV-NP, Cy2, green) was lacking in non-infected DH82 cells (A). Persistently CDV-infected DH82 cells exhibited an immunolabeling for this marker in ≥ 95% of cells (B). Nuclei were labeled with bisbenzimide (blue). Bar=20µm

**Suppl. Fig. 3:**

Comparison of the cumulative population doubling of non-infected and persistently CDV-infected DH82 cells. The persistent infection state of CDV-Ond in DH82 cells did not influence proliferation as demonstrated by the lack of significance (p=0.6347) between the cell population doubling over 14 weeks.

**Suppl. Fig. 4:**

Graphical outline of the morphological cellular changes in non-infected and persistently CDV-infected DH82 cells. Initially, non-infected DH82 cells (A) displayed a high percentage of round cells, which decreased during the first 24h followed by a constant increase of cells with this morphology. In contrast, DH82Ond pi cells (B) showed a constant high percentage of round cells during the first 48 h followed by a transient decrease. The percentage of triangle- and cigar-shaped cells slightly increased during the first 48 h followed by a reversion to the initial amount in non-infected (A) cells. In DH82Ond pi cells, the percentage of triangle- and cigar-shaped cells displayed a pronounced decrease starting at day 3 (B). In non-infected and DH82Ond pi cells, the percentage of cells with a slender morphology remained constant over time (A,B).

**Suppl. Fig. 5:**

Morphology of non-infected and persistently CDV-infected cell cultures. Non-infected DH82 cells (A) displayed a high pleomorphism while round cells predominated within the DH82Ond pi cell population (B).

**Suppl. Fig. 6:** 3D reconstructions from double labeling immunofluorescence of non-infected DH82 and DH82Ond pi cells stained respectively for E-cadherin / WGA (A-C) and cytokeratin 8 / WGA (D-F).

(A) 2D pictures of non-infected DH82 cells from z-stacks obtained with laser scanning confocal microscopy. The single channel views are: nuclei in blue, stained with bisbenzimide (A1); E-cadherin in green, stained with Alexa Fluor 488 (A2); and WGA in red, conjugated with Alexa Fluor 633 (A3). In addition, the picture shows a merged 3 channels view (A4).

(B) Top view of the 3D reconstruction obtained from the z-stack in (A), with the total cell volume represented by WGA in red. E-cadherin was characterized by a variably extended, membranous to cytoplasmic expression.

(C) Section-view of the 3D reconstruction in (B), showing the model sectioned along the green and the red planes to better display the E-cadherin expression (green) within the cell volume (red). E-cadherin showed a membranous to cytoplasmic localization (C, insert), frequently intermingling with the cell membrane.

Each arrow represent the same cell from the z-stack picture to the 3D section-view. 3D reconstruction of non-infected DH82 cells double immunolabeling for E-cadherin-WGA was obtained by 65 z-stack frames (0.13 µm steps).

(D) 2D pictures of DH82Ond pi cells from z-stacks obtained with laser scanning confocal microscopy. The single channel views are: nuclei in blue, stained with bisbenzimide (D1); cytokeratin 8 in green, stained with Alexa Fluor 488 (D2); and WGA in red, conjugated with Alexa fluor 633 (D3). In addition, the picture shows a merged 3 channels view (D4).

(E) Top view of the 3D reconstruction obtained from the z-stack in (D), with the total cell volume represented by WGA in red. Cytokeratin 8 expression was often detected on the cell surface, frequently arranging in variably sized aggregates.

(F) Section-view of the 3D reconstruction in (E), showing the model sectioned along the green and the red planes to better display cytokeratin 8 expression (green) within the cell volume (red). the protein showed a membranous to cytoplasmic expression characterized by variably-sized, frequently sub-membranous immunopositive aggregates.

Each arrow represents the same cell from the z-stack picture to the 3D section-view. 3D reconstruction of DH82Ond pi cells double immunolabeling for cytokeratin 8 / WGA was obtained by 82 z-stack frames (0.13 µm steps).

**Suppl. Fig. 7:** Additional pictures of immunofluorescence for epithelial markers in non-infected (A, C, E) and persistently CDV-infected DH82 cells (B, D, F). Nuclei were labeled with bisbenzimide (blue). Staining for β-catenin (Cy3, red) in non-infected (A1-3) and persistently CDV-infected DH82 cells (B1-3). Staining for E-cadherin (Cy2, green) in non-infected (C1-3) and persistently CDV-infected DH82 cells (D1-3). Staining for cytokeratin 8 (Cy3, red) in non-infected (E1-3) and persistently CDV-infected DH82 cells (F1-3). Non-infected DH82 (A1-3) and DH82Ond pi (B1-3) cells immunolabeled for β-catenin showed a variable membranous to cytoplasmic (A2, arrow) and diffuse cytoplasmic (B1, encircled) protein expression. E-cadherin immunopositive cells in non-infected controls (C1-3) and DH82Ond pi (D1-3) displayed a membranous to cytoplasmic expression (C1, arrow), and a diffuse (C2, encircled) or focal (D2, arrowhead) cytoplasmic expression of this protein. Cytokeratin 8 immunostaining in non-infected DH82 cells frequently revealed a focal cytoplasmic (E2, arrowhead) expression while an only occasional membranous to cytoplasmic (E3, arrow) expression was detected (E1-3). Persistently CDV-Ond infected cells displaied a membranous to cytoplasmic expression (F3, arrow) of cytokeratin 8 (F1-3). Bar=20µm.

**Suppl. Fig. 8:** Additional pictures of immunofluorescence for mesenchymal markers in non-infected (A, C) and persistently CDV-infected DH82 cells (B, D). Nuclei were labeled with bisbenzimide (blue). Staining for N-cadherin (Cy3, red) in non-infected (A1-3) and persistently CDV-infected DH82 cells (B1-3). Staining for vimentin (Cy2, green) in non-infected (C1-3) and persistently CDV-infected DH82 cells (D1-3). Non-infected DH82 (A1-3) cells immunolabeled for N-cadherin showed more frequently a membranous protein expression (A1, arrow) while in DH82Ond pi cells (B1-3) the marker expression ranged from “membranous to cytoplasmic” (B2, encircled) to purely membranous (B2, arrow). Vimentin immunopositive cells in non-infected controls (C1-3) displayed a diffuse cytoplasmic expression within spindle shaped cells (C2, encircled) while DH82Ond pi (D1-3) cells often showed a focal cytoplasmic expression within round cells (D2, arrowhead). Bar=20µm.
